# Engineering amino acid residues of pentacyclic triterpene synthases for improving the activity

**DOI:** 10.1007/s00253-024-13030-8

**Published:** 2024-02-07

**Authors:** Hao Guo, Tongtong Chen, Hanrong Zhu, Huiyan Wang, Yi-Xin Huo

**Affiliations:** 1 Key Laboratory of Molecular Medicine and Biotherapy, School of Life Science, Beijing Institute of Technology, No. 5 South Zhongguancun Street, Beijing, 100081 China; 2 Present Address: Beijing Institute of Technology (Tangshan), Translational Research Center, Hebei, China

**Keywords:** Yeast, Pentacyclic triterpene synthases, Catalytic activity, Baccharenyl cations

## Abstract

**Abstract:**

Pentacyclic triterpenoids exhibit a wide range of biological activities which have wide applications in the food, cosmetics, and pharmaceutical industries. High-performance chassis strains have been developed for the production of various pentacyclic triterpenoids, e.g., lupane-type and oleanane-type triterpenoids. The production of common pentacyclic triterpenes and their derivatives is limited by the poor activity of typical pentacyclic triterpene synthases (PTSs). However, a general strategy applicable to typical PTSs is still lacking. As typical pentacyclic triterpenes are derived from the baccharenyl cation, engineering the non-active-site residues in the MXXXXR motif might be beneficial for the catalytic efficiencies of typical PTSs by the stabilization of the baccharenyl cation. Here, we develop a general strategy for improving the activity of typical PTSs. As a proof of concept, the activity of three PTSs such as lupeol synthase, β-amyrin synthase, and α-amyrin synthases was significantly increased up to 7.3-fold by site-directed saturation mutagenesis. This strategy could be applied to improve the activity of various typical PTSs.

**Key points:**

*• The strategy could be applied to typical PTSs for improving the activity.*

*• The catalytic activity of typical PTSs was significantly increased.*

**Supplementary Information:**

The online version contains supplementary material available at 10.1007/s00253-024-13030-8.

## Introduction

**Fig. 1:**
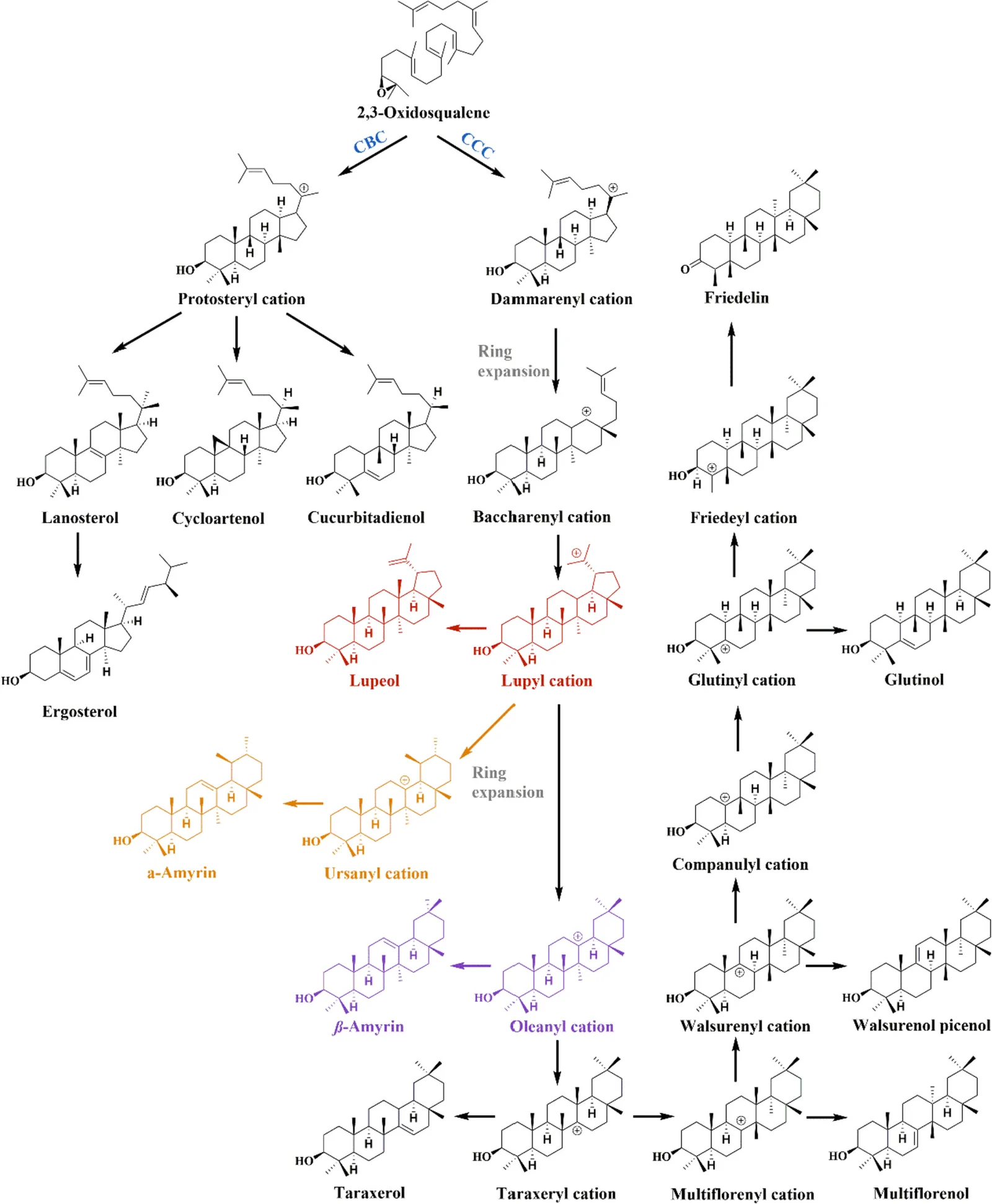
Biosynthesis of triterpenes and sterols catalyzed by 2,3-oxidosqualene cyclases. 2,3-Oxidosqualene is the common precursor for the biosynthesis of triterpenes and sterols. Pentacyclic triterpenes are mainly derived from the baccharenyl cation in the “chair–chair–chair” conformation. The baccharenyl cation was generated by the dammarenyl cation via the ring expansion, which leads to the formation of a large diversity of pentacyclic triterpenoids. Sterols like cycloartenol and lanosterol are mainly generated by the protosteryl cation in the “chair–boat–chair” conformation

Triterpene synthases catalyze the cyclization of 2,3-oxidosqualene to triterpenes and sterols by diverse cationic intermediates. 2,3-Oxidosqualene is mainly cyclized to triterpenes based on the dammarenyl cation via the chair-chair-chair (C–C-C) conformation whereas sterols and minor triterpenes are derived from the protosteryl cation through the chair-boat–chair (C-B-C) conformation (Chin et al. 1973; Lou et al. 1991; Thimmappa et al. 2014). The dammarenyl cation could directly generate dammarane-type tetracyclic triterpenoids which undergo the ring expansion to yield the baccharenyl cation. Typical pentacyclic triterpenoids such as lupane, oleanane, ursane-type, and friedelane-type are derived from the baccharenyl cation while rare pentacyclic triterpenoids could be generated via the protosteryl cation (Fig. 1) (Xu et al. 2004). These cyclized skeletons could be further decorated by cytochrome P450 monooxygenases (P450s), cytochrome P450 reductases (CPRs), and UDP-dependent glycosyltransferases (UGTs), thereby generating numerous pentacyclic triterpenoids. These baccharenyl cation-derived pentacyclic triterpenoids showed various biological activities. For instance, lupeol, α-amyrin, and β-amyrin exhibit various biological activities including antitumor (Barros et al. 2011), anti-inflammatory (Lima et al. 2014), anxiolytic (Aragão et al. 2006), neuroprotective (Wu et al. 2020b), and hepatoprotective effects (Oliveira et al. 2004; Saleem 2009). Furthermore, friedelin was reported to be the precursor of antitumoral quinonemethide triterpenoids (Corsino et al. 2000).

Currently, the production of various triterpenoids was mainly achieved by direct extraction from plant tissues (Lima et al. 2015; Lu et al. 2018). However, the low abundance and unstable supply limited the sustainable production of triterpenoids (Sun et al. 2019). Microbial synthesis of triterpenoids is an attractive and promising alternative because of the short producing cycle and land-saving culture conditions (Li et al. 2019; Noushahi et al. 2022). Well-studied microbes such as *Saccharomyces cerevisiae* and *Yarrowia lipolytica* are good candidate hosts for microbial production of various triterpenoids as they provide the precursor 2,3-oxidosqualene by its endogenous mevalonate (MVA) pathway and membrane structure for the expression of corresponding enzymes P450s and CPRs involved in triterpenoid biosynthetic pathways (Cho et al. 2022; Guo et al. 2020; Zhao and Li 2018).

Metabolic engineering strategies have been employed to improve the production of triterpenoids in microbes (Li et al. 2016). By enhancing the mevalonate (MVA) pathway and triterpenoid biosynthetic pathway, the yield of dammarane-type triterpenoids in *S*. *cerevisiae* has reached the 10 g/L scale (Wang et al. 2019). Furthermore, several high 2,3-oxidosqualene-producing yeast strains have been successfully engineered to produce various triterpenoids by modifying the endogenous lanosterol synthase (Erg7p) (Guo et al. 2022a, b). Based on these strains, the production of various triterpenoids was increased to above 5 g/L in the bioreactor (Guo et al. 2022a). However, the production of typical pentacyclic triterpenoids is relatively lower than dammarane-type triterpenoids when similar strategies were applied to the same chassis (Guo et al. 2020; Huang et al. 2019; Wang et al. 2019). Hence, the major challenge for improving the production of typical pentacyclic triterpenoids is the poor catalytic activity of corresponding pentacyclic triterpene synthases (PTSs), and the strategy for improving the activity of typical PTSs is still lacking.

**Fig. 2:**
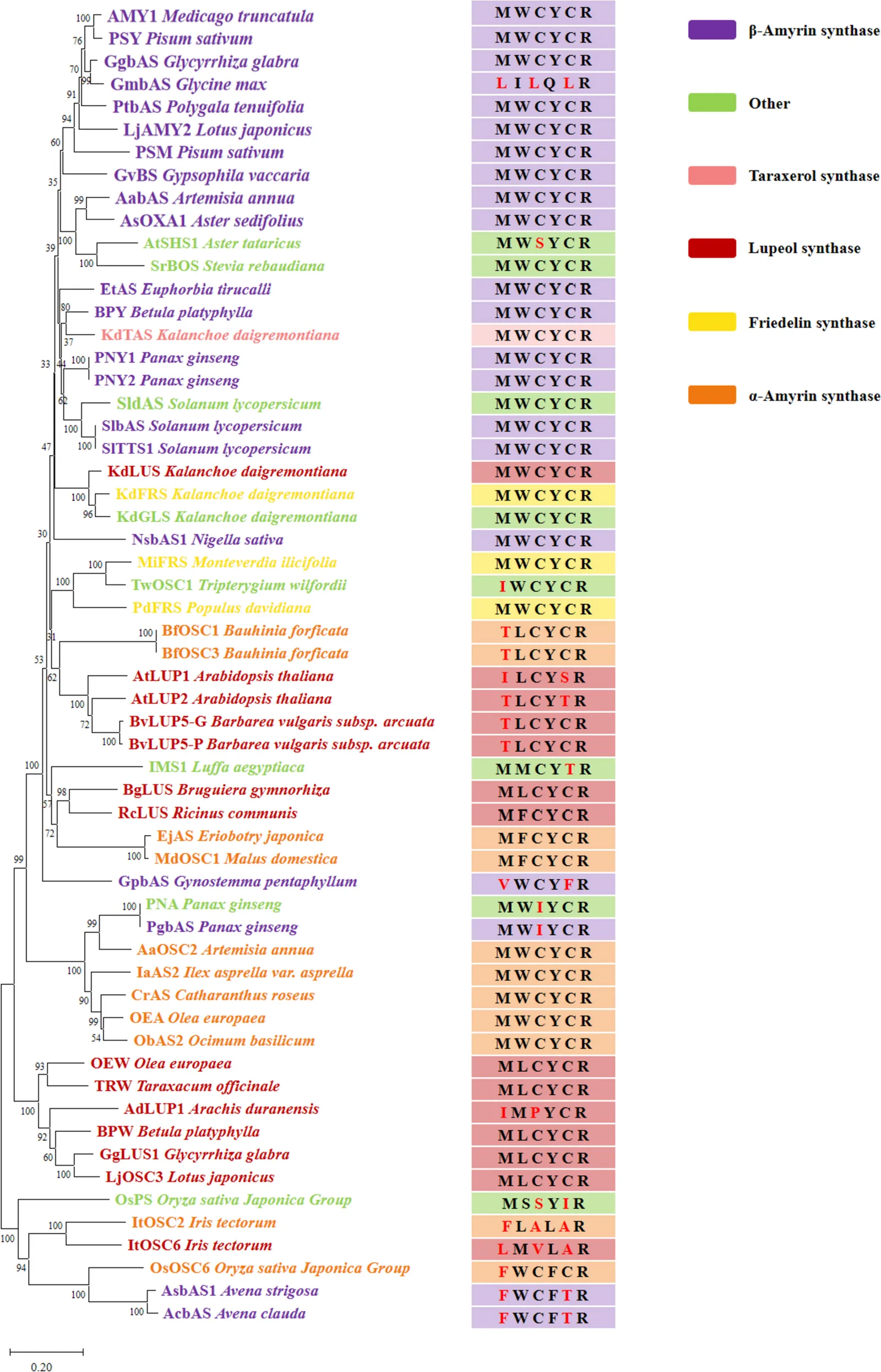
Phylogenetic analysis combined with the MXXXXR motif of characterized pentacyclic triterpene synthases (PTSs). Sequences were selected from GenBank based on their authentication in the literature (unless otherwise indicated) which was built using the neighbor-joining method. Distances were computed using the Poisson correction method. The phylogenetic tree was constructed using 1000 bootstrap replicates. The percentage of trees in which the associated taxa clustered together was shown next to the branches. PTSs could be functionally grouped into lupeol synthases (red), β-amyrin synthases (purple), multifunctional α-amyrin synthases (orange), taraxerol synthase (pink), and friedelin synthase (yellow). Evolutionary analyses were conducted in the software MEGA11. The scale bar indicates 0.2 amino acid substitutions per site

The key amino acid residue motifs such as DCTAE and MXXXXR motifs have been characterized in 2,3-oxidosqualene cyclases (Chen et al. 2021; Wang et al. 2022). The MXXXXR motif is generally characterized as an essential characteristic of typical PTSs, and the active sites of the MXXXXR motif are considered highly conserved (Fig. 2). In the case of β-amyrin synthase from *Euphorbia tirucalli* (EtbAS), the active-site residues Trp257 and Tyr259 involved in the M^256^WCYCR^261^ motif were reported to stabilize the oleanyl cation and the baccharenyl cation via cation-π interaction, respectively (Ito et al. 2016). For lupeol synthases from *Olea europaea* (OEW), a previous study demonstrated that the active-site residues Leu256 and Tyr258 involved in the M^255^LCYCR^260^ might stabilize the lupenyl cation (Kushiro et al. 2000). Modification of these active sites could affect the cation-π interaction, thereby leading to altered end products. In addition, the Trp260 residue in the α-amyrin synthase of *Camellia sasanqua* was proposed to stabilize the ursanyl cation through cation − π interactions and CH − π interactions (Huang et al. 2022). In contrast, the non-active-site residues such as Met255, Cys257, and Cys259 in the MXXXXR motif of EtbAS are not highly conserved in typical PTSs. Engineering the residue such as Cys262 in the M^258^WCYCR^263^ motif of the β-amyrin synthase from *Panax ginseng* did not alter the product selectivity (Kushiro et al. 2000). However, the influence of these residues on the catalytic activity has not been characterized to date. Recently, two PTSs in *Iris tectorum* ItOSC2 and ItOSC6 have been characterized to possess non-canonical motifs F^255^LALAR^260^ and L^255^MVLAR^260^, respectively (Wu et al. 2020a). This study also demonstrated that these non-active-site residues might function as second shell residues in the non-canonical motif and mutation of these non-active sites could influence the catalytic activity (Wu et al. 2020a). As the biosynthesis of the typical pentacyclic triterpenoids is derived from the baccharenyl cation, engineering the non-active-site residues in the MXXXXR motif of the PTSs might be a general strategy for improving the catalytic activities.

In this study, we employed the above strategy to improve the activity of typical PTSs. Taking lupeol synthase, β-amyrin synthase, and α-amyrin synthase as examples, we first investigated the function of the non-active-sites residues in the MXXXXR motif by the computational model. We further investigated the influence of these residues on the catalytic activity of typical PTSs by site-directed saturation mutagenesis, which was characterized by the production of the corresponding products. Finally, we investigated whether the combinatorial mutants based on the beneficial mutations could influence the catalytic activity of typical PTSs. This study provides a general and novel target that could improve the activity of typical PTSs.

## Materials and methods

### Chemicals, strains, and medium

All chemicals and reagents used were obtained from the manufacturer Sinopharm Chemical Reagent Co., Ltd. 601 (Beijing, China). Standards of lupeol (purity ≥ 98%), β-amyrin (purity ≥ 95%), and α-amyrin (purity ≥ 95%) were supplied from Shanghai yuanye, Bio-Technology Co., Ltd. (China). Primers were synthesized by GENEWIZ Bio Inc. (Suzhou, China). Strain SP1 derived from *S*. *cerevisiae* BY4742-trp was used as the initial strain for the expression of all PTSs in this study. Gene cloning was carried out in *Escherichia coli* XL10-gold. All plasmids and strains used in this study are listed in Table S1 and S2, respectively. *S*. *cerevisiae* strains were cultured at 30 °C whereas *E*. *coli* strains were grown at 37 °C in an incubator shaker at 200 rpm. YPD medium consists of 10 g/L yeast extract, 20 g/L peptone, and 20 g/L glucose (20 g/L agar, only for solid medium). Synthetic complete (SC) medium consists of 6.7 g/L yeast nitrogen base without amino acids and with ammonium sulfate, 20 g/L glucose, yeast synthetic drop-out medium supplements without uracil, histidine, leucine, and tryptophan (20 g/L agar, only for solid medium). LB medium consists of 10 g/L tryptone, 5 g/L yeast extract, and 10 g/L NaCl (20 g/L agar, only for solid medium). For *E. coli*, 50 µg/mL of ampicillin was routinely supplemented in broth or solid media, respectively.

### Plasmid construction

Gibson assembly technique was used for the construction of all plasmids. All plasmids and strains used in this study are described in Table S1 and S2, respectively. Primers used in this study are shown in Table S3. GgbAS (encoding β-amyrin synthase from *Glycyrrhiza glabra*, GenBank: AB037203), OEW (encoding lupeol synthase from *Olea europaea*, GenBank no. AB025343), and MdOSC1 genes (encoding α-amyrin synthase from *Malus* × *domestica*, GenBank no. FJ032006.1) were generated by PCR-based gene synthesis, and the resulting fragments were ligated into the pCfB255, resulting in the plasmids p056-LOXP-GgbAS1-URA, p056-LOXP-OEW-URA, and p056-LOXP-MdOSC1-URA. The GgbAS, OEW, and MdOSC1 genes were expressed under the control of a constitutive *P*_*PGK1*_ promoter and the *T*_*ADH1*_ terminator. To mutate the GgbAS, OEW, and MdOSC1 genes, primers containing corresponding mutations were designed for PCR amplification. Plasmids p056-LOXP-GgbAS1-URA, p056-LOXP-OEW-URA, and p056-LOXP-MdOSC1-URA were used as the template for PCR. To achieve multi-site-directed mutagenesis, plasmids p056-LOXP-GgbAS1-URA, p056-LOXP-OEW-URA, and p056-LOXP-MdOSC1-URA containing the single-site mutants were used as a template for PCR amplification. PrimerSTAR DNA polymerase was employed to amplify genes and mutants. The resulting linear fragments were chemically transformed into *E*. *coli* XL10-gold for construction of target plasmids. All mutant genes were verified by DNA sequencing. The genes of GgbAS, OEW, and MdOSC1 were synthesized by approximately 60 bp oligonucleotides with 20 bp overlaps.

### Strain construction

The corresponding plasmids were transformed into strain SP1 for generating the desired strains. The standard lithium acetate method was applied to transform yeast cells (Daniel Gietz and Woods 2002). The desired transformants of *S*. *cerevisiae* were selected on synthetic complete (SC) media with appropriate amino acids or uracil. Before the transformation, the expression cassette of genes in each plasmid was achieved by PCR amplification and then purified from agarose gel. The desired transformants were verified by colony PCR and sequencing. The PCR mixtures contained 0.1 mM each of the four deoxynucleoside triphosphates (dNTPs), 20 ng of the plasmid template, 2 × buffer, 0.1 µM of each primer, 2 U of Phanta MAX Super-Fidelity DNA Polymerase, and double-distilled water to a final volume of 50 µL. The PCR conditions were 95 °C for 3 min, 30 cycles of 95 °C for 10 s, 56 °C for 30 s, 72 °C for 3 min, and a final extension at 72 °C for 30 min.

### Shake flask cultivation

Pre-cultures were prepared by inoculating a single colony from the fresh agar plate. To prepare the shake flask cultivation, pre-cultures were transferred into the main culture to an initial OD_600_ of 0.4. All the main culture were grown in 250-mL shake flasks containing 25 mL (10% (v/v) of the flask). The cultures were incubated at 30 °C and 220 rpm in a rotary shaker for 72 h. Samples were regularly taken for analysis of various triterpenes and measurement of optical densities. All optical densities at 600 nm (OD_600_) were monitored by using the spectrophotometer. Cell biomass was calculated with an OD_600_/dry cell weight conversion factor of 0.21 g DCW/L.

### Homology modeling and bioinformatics analysis

The protein structure models of the GgbAS, OEW, and MdOSC1 were predicted by using the online bioinformatics tool, iterative threading assembly refinement (I-TASSER). Each model with the highest C-score was chosen for molecular docking. The molecular docking was performed using AutoDock 1.5.6 with the corresponding substrate (lupeol, PubChem CID: 259,846; β-amyrin, PubChem CID: 73,145; α-amyrin, PubChem CID: 73,170) as the ligand. The grid maps were calculated by the AutoGrid program. The box size was set to enveloping ligands. The active sites in the motifs MXXXXR and DCTAE of GgbAS, OEW, and MdOSC1 were kept in the center of the grid box with an 80 × 80 × 80-point. The grid spacing was set to 0.375 Å. The Lamarck’s genetic algorithm (LGA) was used to optimize the conformation of lupeol, α-amyrin, or β-amyrin in the binding pocket. The PyMOL viewer was utilized to check the interaction between key amino acid residues and ligands in the models.

### Analysis of the triterpenes

UHPLC system was applied for the quantification of various triterpenes. A C18 column was used for chromatographic separation. The chromatographic conditions were applied as described previously (Czarnotta et al. 2017). The chromatographic conditions were as follows: 10 µL of the sample was used for measurement; the column oven temperature was set to 40 ℃. Acetonitrile (ACN), methanol, and milli-Q water with 0.2% (v/v) formic acid were used as the mobile phase at a flow rate of 1 mL/min. The ratio of acetonitrile, methanol, and milli-Q water with 0.2% (v/v) formic acid was set to 90:9:1 for 1 min after injection. The total run time was 23 min. For calibration, standard curves of lupeol, β-amyrin, and α-amyrin were prepared with concentrations ranging from 1 to 500 mg/L. The samples were prepared as previous procedures with some modifications. A 500 µL cell suspension aliquots were taken from the shake flask culture and transferred into a 2-mL screwed cap tube. Glass beads (250 µL, 0.5 mm), 1 M HCl (50 µL), and hexane (500 µL) were added and homogenized for 5 min in a Mini-BeadBeater. The initial homogenate was clarified by centrifugation at 13,000 rpm for 15 min at room temperature. The upper hexane layer (300 µL) was evaporated and resuspended in 100 µL methanol. The sample was injected directly into the UHPLC system (Figure S6).

## Results

### Engineering lupeol synthase for increasing lupeol production

To rapidly demonstrate the changes in the catalytic activity of various PTSs, a squalene-producing strain SP1, previously constructed based on *S*. *cerevisiae* BY4742-Trp, was used as the chassis to investigate the activity of typical PTSs. One copy of the gene encoding the target PTS was integrated into the genome of the chassis to ensure constitutive expression throughout the cultivation. To assess the validity of our approach, saturation mutagenesis was applied to three non-active sites in the MXXXXR motif of each PTS. The catalytic activity of the mutant and wild-type PTSs was characterized by the titer and specific titer of the corresponding pentacyclic triterpene in the strain.

**Fig. 3:**
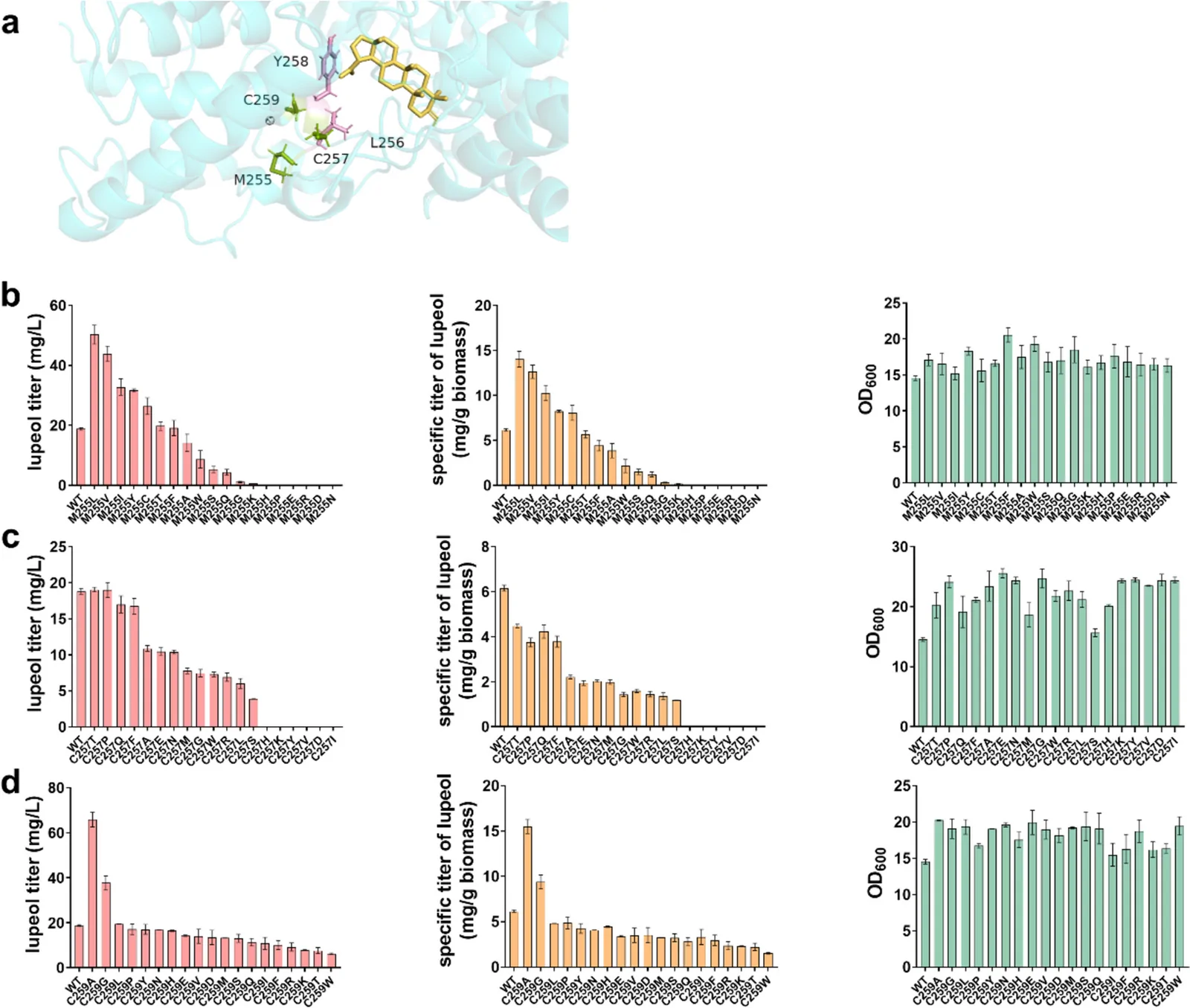
Modification of the non-active-site residues of lupeol synthase from *Olea europaea* (OEW). **a** Molecular docking model of OEW and lupeol using PyMOL software. **b** Influence on the titers of lupeol, specific titer, and cell biomass when saturation mutagenesis was applied on the sites Met255 (**b**), Cys257 (**c**), and Cys259 (**d**) of OEW

Lupeol is derived from the lupenyl cation which is first generated through the ring expansion of the baccharenyl cation. Lupeol synthase has been identified from numerous plants such as *Arabidopsis thaliana*, *Betula platyphylla*, *Bruguiera gymnorrhiza*, and *Olea europaea* (Fig. 2). The previous report demonstrated that strain harboring lupeol synthase from *Olea europaea* (OEW) could generate higher titer of lupeol than that of strain harboring most of other lupeol synthases (Guo et al. 2022a). Here, we first investigated whether engineering the non-active-site residues in the MXXXXR motif could influence the activity of OEW. Molecular docking between the receptor OEW and the ligand lupeol was employed to predict the role of the non-active-site residues Met255, Cys257, and Cys259 of the MXXXXR motif in the OEW. The best-docked model was visualized with the PyMOL viewer. As shown in Fig. 3a and Fig. S1, the non-active-site and active-site amino acid residues were labeled in pink and green, respectively. The lupeol was labeled in yellow. The residue Tyr258 is located near the lupeol. The residues Met255, Cys257, and Cys259 are spatially proximal to the residues Leu256 and Tyr258 which might function as the shell residues. The results of the prediction model suggested that the non-active-site residues might function as the shell residues surrounding the active sites of Leu256 and Tyr258.

Furthermore, site-directed saturation mutagenesis was employed to engineer the aforementioned sites in the MXXXXR motif. As shown in Fig. 3b, strain SP1 harboring OEW^M255L^, OEW^M255V^, OEW^M255I^, OEW^M255Y^, and OEW^M255C^ displayed higher titer and specific titer of lupeol compared with these of the wild-type OEW after 72 h cultivation. To be noted, the strain harboring OEW^M255L^ produced approximately 50 mg/L and 14 mg/g biomass, which are 2.7-fold and 2.3-fold higher than these of the strain harboring the wild-type OEW (Fig. 3b). For the strain SP1 carrying OEW^M255A^, OEW^M255W^, OEW^M255S^, OEW^M255Q^, OEW^M255G^, and OEW^M255K^, the titer and specific titer of lupeol were significantly decreased. The production of lupeol was not detected in the strain SP1 harboring OEW^M255H^, OEW^M255P^, OEW^M255E^, OEW^M255R^, OEW^M255D^, and OEW^M255N^. The OD_600_ of strain SP1 harboring the mutant OEW did not show a significant change compared with that of the wild-type OEW. These results demonstrated that modification of the Met255 could affect the catalytic activity of OEW. Moreover, the five mutants showed higher activity than that of the wild-type OEW.

We then investigated whether the modification of Cys257 and Cys259 in the MXXXXR motif could influence the activity of OEW. To this end, single-site saturation mutagenesis was applied to Cys257 and Cys259. For the Cys257, strain SP1 harboring OEW^C257X^ did not show an increase in the titer and specific titer of lupeol despite that the OD_600_ of these mutants variably increased (Fig. 3c). Moreover, the production of lupeol in the strain SP1 harboring OEW^C257H^, OEW^C257K^, OEW^C257Y^, OEW^C257V^, OEW^C257D^, and OEW^C257I^ was not detected. For the Cys259, most mutants lowered the production of lupeol compared with that of the strain SP1 harboring wild-type OEW, and the strain SP1 harboring OEW^C257X^ showed similar cell biomass. However, strain SP1 harboring OEW^C259A^ and OEW^C259G^ accumulated 65 mg/L and 37.7 mg/L lupeol with the specific titer of 15.5 mg/g biomass and 9.4 mg/g biomass, respectively, which displayed a 3.5-fold and twofold increase in the production of lupeol compared with that of the strain SP1 harboring the wild-type OEW (Fig. 3d). These results demonstrated that modification of Cys257 and Cys259 could affect the activity of OEW, and substitution of Cys259 with A and G could significantly improve the activity of OEW.

### Engineering β-amyrin synthase for increasing β-amyrin production

**Fig. 4:**
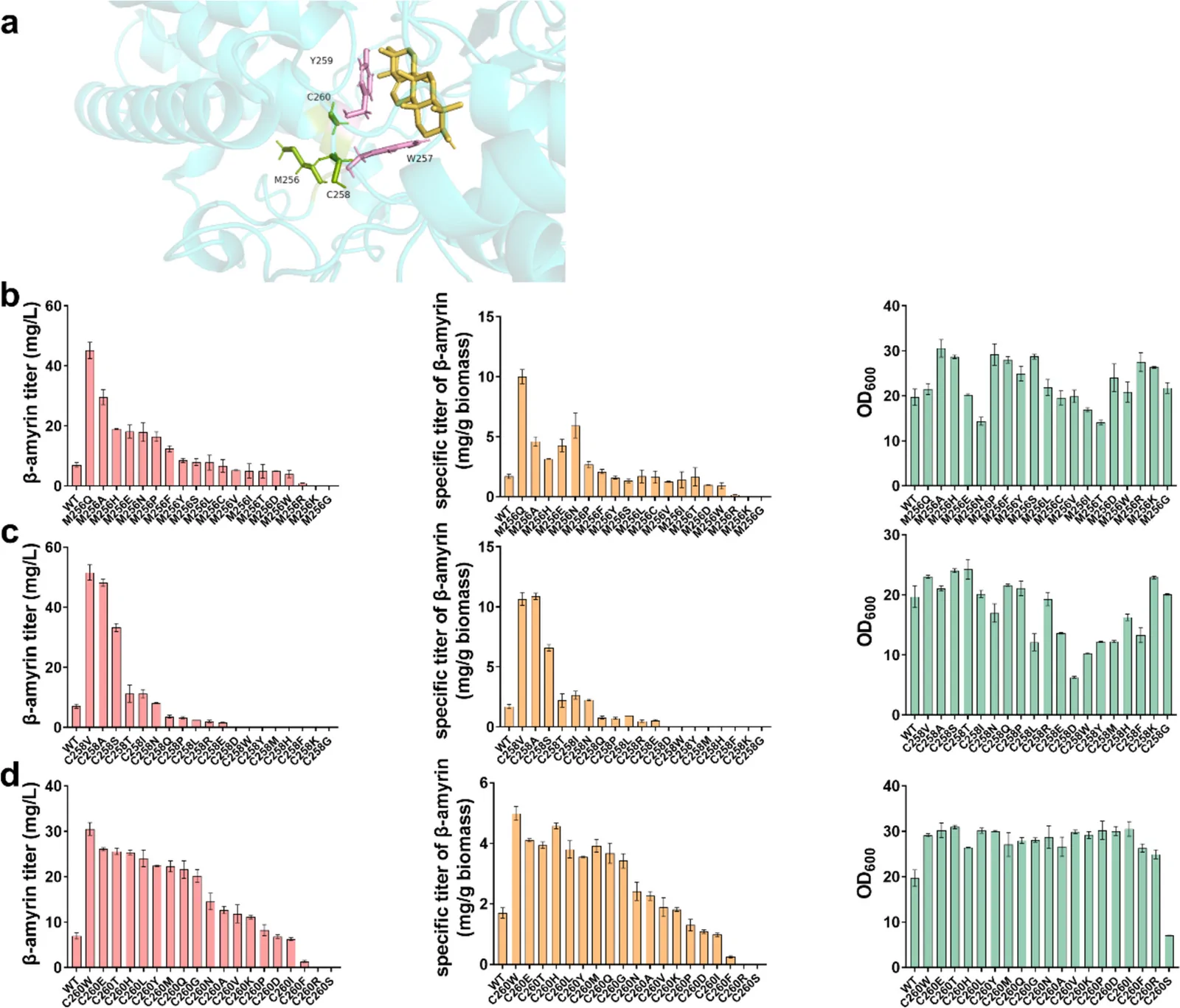
Modification of the non-active-site residues of β-amyrin synthase from *Glycyrrhiza glabra* (GgbAS). **a** Molecular docking model of GgbAS and β-amyrin using PyMOL software. **b** Influence on the titers and specific titer of β-amyrin as well as cell biomass, when saturation mutagenesis was applied on the sites Met256 (**b**), Cys258 (**c**), and Cys260 (**d**) of GgbAS

To demonstrate its applicability, such a strategy was applied to β-amyrin synthase. β-Amyrin is the direct deprotonation product of the oleanyl cation which is formed by further ring expansion of the lupenyl cation via the proton transfer of the intermediate germanicyl cation. β-Amyrin synthase is one of the most common PTSs, widely distributed in plants (Fig. 2). β-Amyrin synthase from *Glycyrrhiza glabra* (GgbAS) was reported to exhibit better catalytic efficiency than that of other β-amyrin synthases from *A*. *annua*, *P*. *sativum*, and *P*. *ginseng* (Dai et al. 2014). Here, GgbAS was used as the representative β-amyrin synthase to verify our strategy. The non-active-site residues Met255, Cys257, and Cys259 in the MXXXXR motif of OEW correspond to Met256, Cys258, and Cys260 in GgbAS. First, the β-amyrin was docked with GgbAS for predicting the role of Met256, Cys258, and Cys260 in the GgbAS. As shown in Fig. 4a and Fig. S2, the residues Trp257 and Tyr259 were spatially proximal to β-amyrin. The residues Met256, Cys258, and Cys260 surrounded the active-site residues Trp257 and Tyr259. This result suggested that the non-active-site residues Met256, Cys258, and Cys260 might function as the shell residues of the active-site residues Trp257 and Tyr259 in the GgbAS.

Single-site saturation mutagenesis was applied to Met256, Cys258, and Cys260. For Met256, strain SP1 harboring GgbAS^M256Q^, GgbAS^M256A^, GgbAS^M256H^, GgbAS^M256E^, GgbAS^M256N^, GgbAS^M256P^, and GgbAS^M256F^ showed higher titer and specific titer of β-amyrin than these of strain SP1 harboring the wild-type GgbAS after 72 h cultivation. Among these beneficial mutants, strain SP1 harboring GgbAS^M256Q^ could produce 45.2 mg/L β-amyrin with the specific titer of 10 mg/g biomass, which are 6.5-fold and 5.9-fold increase compared with that of strain SP1 harboring the wild-type GgbAS. Strain SP1 harboring GgbAS^M256N^ showed a slight decrease in the cell biomass. Furthermore, strain SP1 harboring GgbAS^M256W^, GgbAS^M256R^, GgbAS^M256K^, and GgbAS^M256G^ displayed a significantly decreased or complete loss in the production of β-amyrin (Fig. 4b). These results demonstrated that the activity of β-amyrin synthase could be improved by the modification of Met256.

In addition, we investigated whether the modification of Cys258 and Cys260 could improve the activity of GgbAS. As shown in Fig. 4c, most of the substitutions at the Cys258 did not lead to an increase in the titer and specific titer of β-amyrin when single-site saturation mutagenesis was applied. However, strain SP1 harboring GgbAS^C258V^, GgbAS^C258A^, and GgbAS^C258S^ produced 51.6 mg/L, and 48.2 mg/L, 33.3 mg/L β-amyrin which were 7.3-fold, 6.8-fold, and 4.7-fold higher titer and specific titer of β-amyrin than these of strain SP1 harboring the wild-type GgbAS. The specific titer of β-amyrin in strain SP1 harboring GgbAS^C258V^, GgbAS^C258A^, and GgbAS^C258S^ reached 10.6, 10.9, and 6.6 mg/g biomass, respectively. Compared with Cys258, more mutants at the site of Cys260 could result in a higher titer and specific titer of β-amyrin. Among these mutants, strain SP1 harboring GgbAS^C260W^ produced 30.6 mg/L β-amyrin with 5 mg/g biomass, which was 4.4-fold and 2.8-fold higher than these of strain harboring wild-type GgbAS. Strain SP1 harboring GgbAS^C260R^ and GgbAS^C260S^ resulted in a significant loss in the production of β-amyrin (Fig. 4d). In addition, the cell growth of most mutants at the site of Cys260 could be dramatically increased. These results demonstrated that mutations on the sites of Cys258 and Cys260 could improve the activity of GgbAS. Hence, our strategy could be applied to β-amyrin synthase for improving the activity.

### Engineering α-amyrin synthase for increasing α-amyrin production

**Fig. 5:**
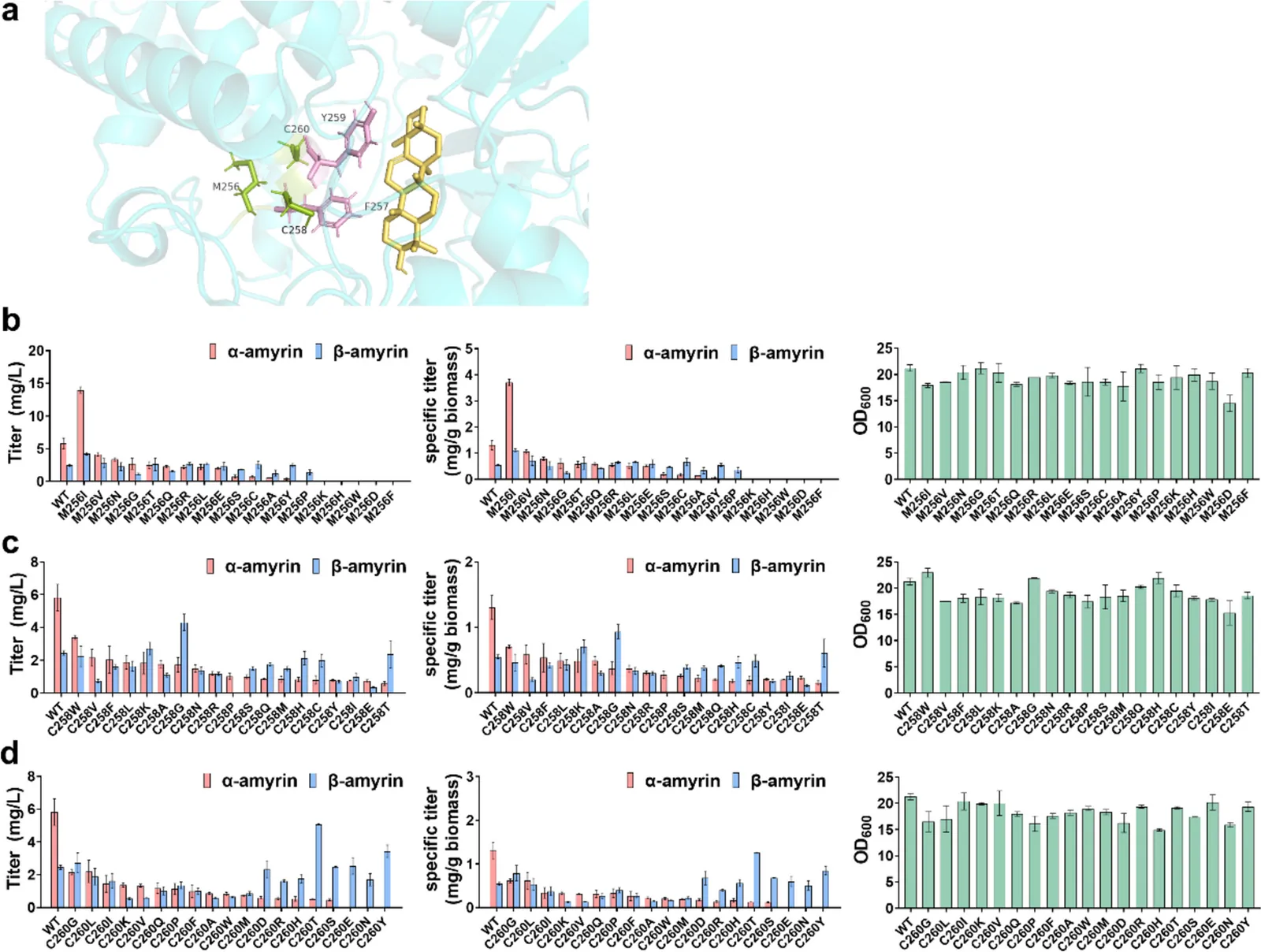
Modification of the non-active-site residues of α-amyrin synthase from *Malus* × *domestica* (MdOSC1). **a** Molecular docking model of MdOSC1 and α-amyrin using PyMOL software. **b** Influence on the titers and specific titer of α-amyrin and β-amyrin as well as cell biomass when saturation mutagenesis was applied on the sites Met256 (**b**), Cys258 (**c**), and Cys260 (**d**) of MdOSC1

As many PTSs have been characterized as multifunctional enzymes in plants, we wished to investigate whether our strategy could be applied to multi-function PTSs. The monofunctional α-amyrin synthase has not been identified in plants to date, which could be utilized as the target PTS for verification of our strategy. α-Amyrin is generated by the ursanyl cation which is derived from the germanicyl cation via the proton transfer (Fig. 2). The previous study reported that the activity of α-amyrin synthase from *Malus* × *domestica* (MdOSC1) was higher than that of other characterized α-amyrin synthases (Yu et al. 2018). Here, we used MdOSC1 as the target PTS for verification of our strategy. The non-active-site residues in the MFCYCR motif of MdOSC1 correspond to Met256, Cys258, and Cys260. First, the role of non-active-site residues Met256, Cys258, and Cys260 was predicted by molecular docking using the MdOSC1 and the α-amyrin. As shown in Fig. 5a and Fig. S3, the active-site residues Phe257 and Tyr259 were adjacent to the ligand α-amyrin whereas the residues Met256, Cys258, and Cys260 surrounding the active-site residues were distant from the ligand α-amyrin. This result suggested that the residues Met256, Cys258, and Cys260 might be the shell residues around the active-site residues Phe257 and Tyr259 in the MdOSC1.

The single-site saturation mutagenesis was applied to Met256, Cys258, and Cys260. Most of the substitutions for Met256 displayed a significant loss in the titer and specific titer of α-amyrin and β-amyrin. Strain SP1 harboring MdOSC1^M256K^, MdOSC1^M256H^, MdOSC1^M256W^, MdOSC1^M256D^, and MdOSC1^M256F^ completely abolished the production of α-amyrin and β-amyrin. Nevertheless, strain SP1 harboring MdOSC1^M256I^ produced about 14 mg/L α-amyrin and 4.2 mg/L β-amyrin with the specific titer of 3.7 mg/g biomass and 1.1 mg/g biomass, which showed a 2.4-fold and 1.8-fold increase in the titer of α-amyrin and β-amyrin, compared with these of the strain SP1 harboring the wild-type MdOSC1 (Fig. 5b). Furthermore, strain SP1 harboring MdOSC1^M256S^, MdOSC1^M256C^, MdOSC1^M256A^, and MdOSC1^M256Y^ produced more β-amyrin than α-amyrin. The α-amyrin to β-amyrin ratios in these mutant strains were compared with the strain harboring wild-type MdOSC1. Strain SP1 harboring MdOSC1^M256P^ produces exclusively β-amyrin instead of α-amyrin and β-amyrin. These results demonstrated that engineering Met256 could change its activity and the ratio of α/β-amyrin.

For Cys258, we did not observe the overall increase in the titer and specific titer of α-amyrin and β-amyrin when single-site saturation mutagenesis was applied. Nevertheless, most mutants such as MdOSC1^C258G^, MdOSC1^C258Q^, MdOSC1^C258M^, MdOSC1^C258H^, and MdOSC1^C258T^ showed the change in the ratio of α/β-amyrin (Fig. 5c). To be noted, strain SP1 harboring MdOSC1^C258P^ exclusively produced α-amyrin after 72 h cultivation. For Cys260, the substitution at the Cys260 showed the changed ratio of α-amyrin/β-amyrin and the overall decrease in the titer and specific titer of α-amyrin. Strain SP1 harboring MdOSC1^C260E^, MdOSC1^C260N^, and MdOSC1^C260Y^ produced β-amyrin instead of α-amyrin and β-amyrin (Fig. 5d). These results demonstrated that our strategy could be applied to multifunction PTSs for improving their activity, and the ratio of mixed products could also be changed when the non-active-site residues of the MXXXXR motif were engineered.

### Combinatorial mutation of beneficial mutations for increasing the production of various PTSs

**Fig. 6:**
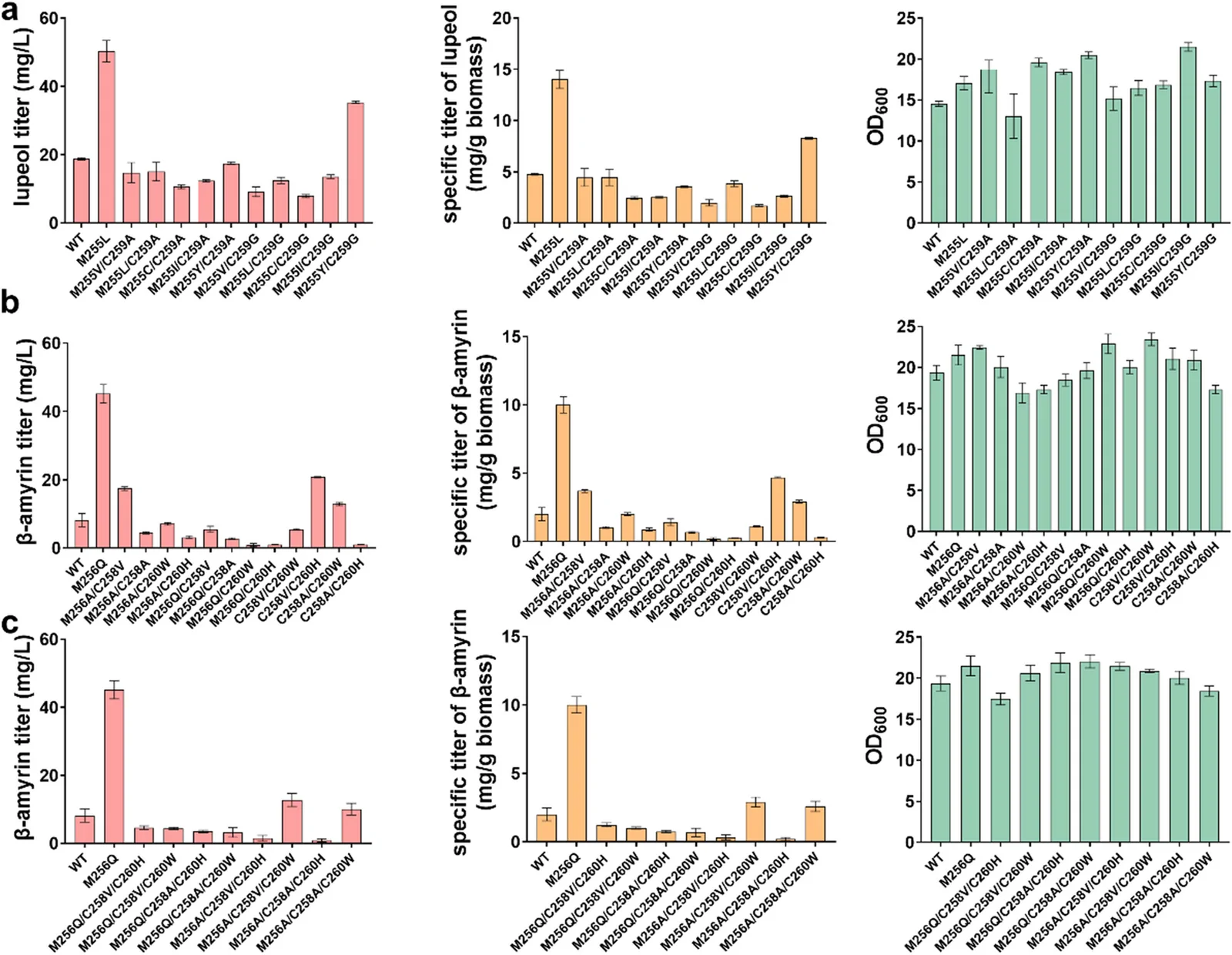
Influence of double and triple mutants on the activity of PTSs. **a** Influence on the titers and specific titer of lupeol as well as cell biomass when double mutants were applied on the sites Met255 and Cys259 of OEW. **b** Influence on the titers and specific titer of β-amyrin as well as cell biomass when double mutants were applied on the sites Met256, Cys258, and Cys260 of GgbAS. **c** Influence on the titers and specific titer of β-amyrin as well as cell biomass, when triple mutants were applied on the sites Met256, Cys258, and Cys260 of GgbAS

Based on the aforementioned beneficial sites of various PTSs, we then investigated whether combinatorial mutagenesis could improve the activity of various PTSs. For OEW, double mutants were constructed based on the significant beneficial single-site mutants of the sites Met255 and Cys259 including M255V, M255L, M255C, M255I, M255Y, C259A, and C259G (Fig. 6a). All strain SP1 carrying these double mutants except OEW^M255YC259G^ accumulated the lower titer and specific titer of lupeol, compared with strain SP1 carrying the wild-type OEW. Strain SP1 carrying OEW^M255YC259G^ achieved an approximately 1.9-fold higher titer of lupeol than SP1 carrying the wild-type OEW. The titer and specific titer of lupeol reached 35 mg/L and 8.27 mg/g biomass, respectively.

For the GgbAS, combinatorial mutagenesis was employed based on the top two beneficial mutants of Met256, Cys258, and Cys260. To be specific, M256A, M256Q, C258A, C258V, C260W, and C260H were chosen to construct twelve double mutants and eight triple mutants, respectively. As shown in Fig. 6b, the strain SP1 carrying GgbAS^M256AC258V^, GgbAS^C258VC260H^, and GgbAS^C258AC260W^ showed a significant increase in the titer and specific titer of β-amyrin where other double mutants lowered the production of β-amyrin. Among these double mutants, the strain SP1 carrying GgbAS^C258VC260H^ produced 20.8 mg/L β-amyrin with a specific titer of 4.7 mg/g biomass. For triple mutants, the strain SP1 carrying GgbAS^M256AC258VC260W^ and GgbAS^M256AC258AC260W^ showed a 1.55-fold and 1.23-fold increase in the production of β-amyrin compared with that of strain SP1 harboring the wild-type GgbAS. The production of β-amyrin in the strain SP1 carrying GgbAS^M256AC258VC260W^ and GgbAS^M256AC258AC260W^ reached 12.8 mg/L and 10.1 mg/L with a specific titer of 2.9 mg/g biomass and 2.6 mg/g biomass (Fig. 6c). For MdOSC1, triple mutants were constructed based on the beneficial single-site mutants in the MdOSC1 and GgbAS. As shown in Figure S4, all the strain SP1 carrying triple mutants accumulated the lower titer and specific titer of α-amyrin, compared with strain SP1 carrying the wild-type MdOSC1. These results indicated that the combinatorial mutagenesis of the beneficial mutant could also influence the activity of PTSs despite that these recombinant mutants showed relatively lower efficiency than that of the single beneficial mutants of PTSs.

## Discussion

In this study, we first reported that engineering the non-active-site residues of the MXXXXR motif could improve their activity, which could be applied to typical PTSs. As a proof of concept, the computational models were used to predict the function of these non-active sites. Several single mutants of OEW, GgbAS, and MdOSC1 exhibiting the higher production of corresponding pentacyclic triterpenes were achieved when site-directed saturation mutagenesis was applied on the non-active-site residues. In addition, double and triple mutants of GgbAS and OEW could also improve the production of corresponding triterpenes. This strategy has the potential to be applied to other non-active-site residues close to the active sites of typical PTSs for improving the activity.

Recently, many strategies have been applied to improve the activity of typical PTSs. Genome mining is a traditional strategy to identify a PTS with high activity in nature (Dale et al. 2020; Wang et al. 2021). Various isoenzymes could be characterized based on the genome and transcriptome data of different plant species. The typical PTSs with high activity could be screened out based on the titer and specific titer of corresponding triterpenes (Dale et al. 2020). However, it is time-consuming to dig up the PTSs with beneficial mutations. In addition, a semi-rational design has been recently applied to improve the activity of a specific PTS. Based on the prediction of the computational model, a mutant MdOSC1^N11T/P250H/P373A^ with high activity was achieved by the single and combinatorial site-direct saturation mutagenesis (Yu et al. 2020). We also investigated whether the mutant N11T/P250H/P373A combined with M255T could further enhance the production of α-amyrin. Nevertheless, the quadruple mutant N11T/P250H/P373A/M255T did not show higher production of α-amyrin than that of the wild-type MdOSC1 (Figure S5).

The active sites of cyclases function as the stabilization of cationic intermediates, which are critical for the catalytic activities and specificities (Hoshino 2017). Most mutations in these active sites would disrupt the cation-π interaction and affect the stability of cationic intermediates, which could decrease the activity of cyclases (Srisawat et al. 2019). For instance, most mutations in the Phe413, Phe728, Trp257, and Tyr259 of EtbAS could decrease its catalytic activity (Ito et al. 2013, 2016). Nevertheless, a previous study suggested that increasing the stability of the cationic intermediates could improve the activity of squalene-hopene cyclase by enhancing the cation-π interaction (Dang and Prestwich 2000). In addition to cyclases, many studies demonstrated that engineering the residues surrounding the active site of enzymes could generate mutated enzymes with improved catalytic activity (Krah et al. 2021). Taken together, engineering the non-active-site’s proximity to active sites of PTSs might influence the cation-π interaction and then led to a change in the catalytic efficiency or specificity of PTSs. For the MdOSC1, we did not observe that engineering Cys258 and Cys260 in the MXXXXR motif could improve the enzyme activity, which might be due to that MdOSC1 is a multi-function PTS. Furthermore, engineering the non-active-site residues in the MXXXXR motif could switch the favor of the ursanyl cation to the oleanyl cation as the non-active-site residues might stabilize the ursanyl cation through cation − π interactions and CH − π interactions (Huang et al. 2022; Wu et al. 2019).

In summary, we developed a novel strategy for improving the activity of PTSs in *S*. *cerevisiae*. Results demonstrated that the titer and specific titer of lupeol, β-amyrin, and α-amyrin were significantly increased up to 7.3-fold when the PTSs harbored corresponding mutations. As a proof of concept, we demonstrated that the activity of typical PTSs could be influenced by engineering the non-active-site residues of the MXXXXR motif, and the activity of three PTSs was successfully improved. This work also provided a new approach for improving the production of pentacyclic triterpenoids.

## Supplementary Information

Below is the link to the electronic supplementary material.

Supplementary file1 (PDF 1199 KB)

## Data Availability

All data generated or analyzed during this study are included in this published article (and its supplementary information files).

## References

[CR1] Aragão G, Carneiro L, Junior A, Vieira L, Bandeira P, Lemos T, de Viana GS (2006) A possible mechanism for anxiolytic and antidepressant effects of alpha-and beta-amyrin from Protium heptaphyllum (Aubl.) March. Pharmacol Biochem Behav 85:827–834 17207523 10.1016/j.pbb.2006.11.019

[CR2] Barros FW, Bandeira PN, Lima DJ, Meira AS, de Farias SS, Albuquerque MR, dos Santos HS, Lemos TL, de Morais MO, Costa-Lotufo LV, Pessoa Cdo Ó (2011) Amyrin esters induce cell death by apoptosis in HL-60 leukemia cells. Bioorg Med Chem 19:1268–1276 21216606 10.1016/j.bmc.2010.12.016

[CR3] Chen K, Zhang M, Ye M, Qiao X (2021) Site-directed mutagenesis and substrate compatibility to reveal the structure–function relationships of plant oxidosqualene cyclases. Nat Prod Rep 38:2261–2275 33988197 10.1039/d1np00015b

[CR4] Chin WJ, Corbett RE, Heng CK, Wilkins AL (1973) Lichens and fungi. Part XI. Isolation and structural elucidation of a new group of triterpenes from *Sticta coronata*, *S. colensoi*, and *S. flavicans*. J Chem Soc Perkin Trans 1437–1446 10.1039/p19730001437 4739023

[CR5] Cho JS, Kim GB, Eun H, Moon CW, Lee SY (2022) Designing microbial cell factories for the production of chemicals. JACS Au 2:1781–1799 36032533 10.1021/jacsau.2c00344 PMC9400054

[CR6] Corsino J, de Carvalho PRF, Kato MJ, Latorre LR, Oliveira OMM, Araújo ARS, Araújo ARS, da Bolzani V, França SC, Pereira AMS, Furlan M (2000) Biosynthesis of friedelane and quinonemethide triterpenoids is compartmentalized in *Maytenus aquifolium* and *Salacia campestris*. Phytochemistry 55:741–748 11190390 10.1016/s0031-9422(00)00285-5

[CR7] Czarnotta E, Dianat M, Korf M, Granica F, Merz J, Maury J, Baallal Jacobsen SA, Förster J, Ebert BE, Blank LM (2017) Fermentation and purification strategies for the production of betulinic acid and its lupane-type precursors in *Saccharomyces cerevisiae*. Biotechnol Bioeng 114:2528–2538 28688186 10.1002/bit.26377

[CR8] Dai Z, Wang B, Liu Y, Shi M, Wang D, Zhang X, Liu T, Huang L, Zhang X (2014) Producing aglycons of ginsenosides in bakers’ yeast. Sci Rep 4:3698 24424342 10.1038/srep03698 PMC3892717

[CR9] Dale MP, Moses T, Johnston EJ, Rosser SJ (2020) A systematic comparison of triterpenoid biosynthetic enzymes for the production of oleanolic acid in *Saccharomyces cerevisiae*. PLoS ONE 15:e0231980 32357188 10.1371/journal.pone.0231980 PMC7194398

[CR10] Dang T, Prestwich GD (2000) Site-directed mutagenesis of squalene–hopene cyclase: altered substrate specificity and product distribution. Chem Biol 7:643–649 11048954 10.1016/s1074-5521(00)00003-x

[CR11] Daniel Gietz R, Woods RA (2002) Transformation of yeast by lithium acetate/single-stranded carrier DNA/polyethylene glycol method. Meth Enzymol 350:87–96 10.1016/s0076-6879(02)50957-5 12073338

[CR12] Guo H, Wang H, Huo YX (2020) Engineering critical enzymes and pathways for improved triterpenoid biosynthesis in yeast. ACS Synth Biol 9:2214–2227 32786348 10.1021/acssynbio.0c00124

[CR14] Guo H, Jacobsen SAB, Walter K, Lewandowski A, Czarnotta E, Knuf C, Polakowski T, Maury J, Lang C, Förster J, Blank LM, Ebert BE (2022a) Triterpenoid production with a minimally engineered *Saccharomyces cerevisiae* chassis. bioRxiv. 10.1101/2022.07.11.499565

[CR13] Guo H, Wang H, Chen T, Guo L, Blank LM, Ebert BE, Huo Y-X (2022b) Engineering critical amino acid residues of lanosterol synthase to improve the production of triterpenoids in *Saccharomyces cerevisiae*. ACS Synth Biol 11:2685–2696 35921601 10.1021/acssynbio.2c00098

[CR15] Hoshino T (2017) β-Amyrin biosynthesis: catalytic mechanism and substrate recognition. Org Biomol Chem 15:2869–2891 28294269 10.1039/c7ob00238f

[CR16] Huang J, Zha W, An T, Dong H, Huang Y, Wang D, Yu R, Duan L, Zhang X, Peters RJ, Dai Z, Zi J (2019) Identification of RoCYP01 (CYP716A155) enables construction of engineered yeast for high-yield production of betulinic acid. Appl Microbiol Biotechnol 103:7029–7039 31309269 10.1007/s00253-019-10004-z

[CR17] Huang L, Hu Y, Huang R, Chen J, Zhang X, Yue J, Feng L, She Y, Ji A, Zheng Y, Liu Z, Zhang R, Duan L (2022) Oxidosqualene cyclases involved in the biosynthesis of diverse triterpenes in *Camellia sasanqua*. J Agric Food Chem 70:8075–8084 35729682 10.1021/acs.jafc.2c03011

[CR18] Ito R, Hashimoto I, Masukawa Y, Hoshino T (2013) Effect of cation–π interactions and steric bulk on the catalytic action of oxidosqualene cyclase: a case study of Phe728 of β-amyrin synthase from *Euphorbia tirucalli L*. Chem Eur J 19:17150–17158 24203491 10.1002/chem.201301917

[CR19] Ito R, Nakada C, Hoshino T (2016) β-Amyrin synthase from *Euphorbia tirucalli* L. functional analyses of the highly conserved aromatic residues Phe413, Tyr259 and Trp257 disclose the importance of the appropriate steric bulk, and cation-π and CH-π interactions for the efficient catalytic action of the polyolefin cyclization cascade. Org Biomol Chem 15:177–188 27942657 10.1039/c6ob02539k

[CR20] Krah A, van der Hoeven B, Mestrom L, Tonin F, Knobel KCC, Bond PJ, McMillan DGG (2021) A second shell residue modulates a conserved ATP-binding site with radically different affinities for ATP. Biochim Biophys Acta - Gen Subj 1865:129766 33069831 10.1016/j.bbagen.2020.129766

[CR21] Kushiro T, Shibuya M, Masuda K, Ebizuka Y (2000) Mutational studies on triterpene synthases: engineering lupeol synthase into β-amyrin synthase. J Am Chem Soc 122:6816–6824

[CR22] Li D, Zhang Q, Zhou Z, Zhao F, Lu W (2016) Heterologous biosynthesis of triterpenoid dammarenediol-II in engineered *Escherichia coli*. Biotechnol Lett 38:603–609 26739962 10.1007/s10529-015-2032-9

[CR23] Li D, Wu Y, Zhang C, Sun J, Zhou Z, Lu W (2019) Production of triterpene ginsenoside compound K in the non-conventional yeast *Yarrowia lipolytica*. J Agric Food Chem 67:2581–2588 30757901 10.1021/acs.jafc.9b00009

[CR24] Lima EM, Nascimento AM, Lenz D, Scherer R, Meyrelles SS, Boëchat GA, Andrade TU, Endringer DC (2014) Triterpenes from the *Protium heptaphyllum* resin-chemical composition and cytotoxicity. Rev Bras Farmacogn 24:399–407

[CR25] Lima AM, Siani AC, Nakamura MJ, D’Avila LA (2015) Selective and cost-effective protocol to separate bioactive triterpene acids from plant matrices using alkalinized ethanol: application to leaves of Myrtaceae species. Pharmacogn Mag 11:470–476 26246721 10.4103/0973-1296.160453 PMC4522832

[CR26] Lou H, Li X, Onda M, Konda Y, Urano M, Harigaya Y, Takayanagi H, Ogura H (1991) Stereochemistry of novel triterpenes from *Cynanchum hancokianum*. Chem Pharm Bull 39:2271–2276

[CR27] Lu C, Zhang C, Zhao F, Li D, Lu W (2018) Biosynthesis of ursolic acid and oleanolic acid in *Saccharomyces cerevisiae*. AIChE J 64:3794–3802

[CR28] Noushahi HA, Khan AH, Noushahi UF, Hussain M, Javed T, Zafar M, Batool M, Ahmed U, Liu K, Harrison MT, Saud S, Fahad S, Shu S (2022) Biosynthetic pathways of triterpenoids and strategies to improve their biosynthetic efficiency. Plant Growth Regul 97:439–454 35382096 10.1007/s10725-022-00818-9 PMC8969394

[CR29] Oliveira FA, Vieira-Júnior GM, Chaves MH, Almeida FR, Florêncio MG, Lima RC Jr, Silva RM, Santos FA, Rao VS (2004) Gastroprotective and anti-inflammatory effects of resin from *Protium heptaphyllum* in mice and rats. Pharmacol Res 49:105–111 14643690 10.1016/j.phrs.2003.09.001

[CR30] Saleem M (2009) Lupeol, a novel anti-inflammatory and anti-cancer dietary triterpene. Cancer Lett 285:109–115 19464787 10.1016/j.canlet.2009.04.033 PMC2764818

[CR31] Srisawat P, Fukushima EO, Yasumoto S, Robertlee J, Suzuki H, Seki H, Muranaka T (2019) Identification of oxidosqualene cyclases from the medicinal legume tree *Bauhinia forficata*: a step toward discovering preponderant α-amyrin-producing activity. New Phytol 224:352–366 31230357 10.1111/nph.16013

[CR32] Sun W, Qin L, Xue H, Yu Y, Ma Y, Wang Y, Li C (2019) Novel trends for producing plant triterpenoids in yeast. Crit Rev Biotechnol 39:618–632 31068012 10.1080/07388551.2019.1608503

[CR33] Thimmappa R, Geisler K, Louveau T, O’Maille P, Osbourn A (2014) Triterpene biosynthesis in plants. Annu Rev Plant Biol 65:225–257 24498976 10.1146/annurev-arplant-050312-120229

[CR34] Wang P, Wei W, Ye W, Li X, Zhao W, Yang C, Li C, Yan X, Zhou Z (2019) Synthesizing ginsenoside Rh2 in *Saccharomyces cerevisiae* cell factory at high-efficiency. Cell Discov 5:5 30652026 10.1038/s41421-018-0075-5 PMC6331602

[CR35] Wang H, Guo H, Wang N, Huo YX (2021) Toward the heterologous biosynthesis of plant natural products: gene discovery and characterization. ACS Synth Biol 10:2784–2795 34757715 10.1021/acssynbio.1c00315

[CR36] Wang P, Wei G, Feng L (2022) Research advances in oxidosqualene cyclase in plants. Forests 13:1382

[CR37] Wu S, Zhang F, Xiong W, Molnár I, Liang J, Ji A, Wang C, Wang S, Liu Z, Wu R, Duan L (2020a) An unexpected oxidosqualene cyclase active site architecture in the *Iris tectorum* multifunctional α-amyrin synthase. ACS Catal 10:9515–9520 34306805 10.1021/acscatal.0c03231 PMC8297885

[CR38] Wu Z-W, Li W-B, Zhou J, Liu X, Wang L, Chen B, Wang M-K, Ji L, Hu W-C, Li F (2020b) Oleanane- and ursane-type triterpene saponins from *Centella asiatica* exhibit neuroprotective effects. J Agric Food Chem 68:6977–6986 32502339 10.1021/acs.jafc.0c01476

[CR39] Wu Z, Xu H, Wang M, Zhan R, Chen W, Zhang R, Kuang Z, Zhang F, Wang K, Gu J (2019) Molecular docking and molecular dynamics studies on selective synthesis of α-amyrin and β-amyrin by oxidosqualene cyclases from *Ilex Asprella*. Int J Mol Sci 20(14):3469 31311103 10.3390/ijms20143469 PMC6678101

[CR40] Xu R, Fazio GC, Matsuda SP (2004) On the origins of triterpenoid skeletal diversity. Phytochemistry 65:261–291 14751299 10.1016/j.phytochem.2003.11.014

[CR41] Yu Y, Chang P, Yu H, Ren H, Hong D, Li Z, Wang Y, Song H, Huo Y, Li C (2018) Productive amyrin synthases for efficient α-amyrin synthesis in engineered *Saccharomyces cerevisiae*. ACS Synth Biol 7:2391–2402 30216049 10.1021/acssynbio.8b00176

[CR42] Yu Y, Rasool A, Liu H, Lv B, Chang P, Song H, Wang Y, Li C (2020) Engineering *Saccharomyces cerevisiae* for high yield production of α-amyrin via synergistic remodeling of α-amyrin synthase and expanding the storage pool. Metab Eng 62:72–83 32841679 10.1016/j.ymben.2020.08.010

[CR43] Zhao Y-J, Li C (2018) Biosynthesis of plant triterpenoid saponins in microbial cell factories. J Agric Food Chem 66:12155–12165 30387353 10.1021/acs.jafc.8b04657

