# Supplementary material for: Engineering amino acid residues of pentacyclic triterpene synthases for improving the activity
Source: Appl Microbiol Biotechnol. 2024 Feb 7;108(1):195. doi: 10.1007/s00253-024-13030-8 (PMC10850208; doi:10.1007/s00253-024-13030-8)
Supplement: Supplementary file 1 — Supplementary file1 (PDF 1199 KB) [file 253_2024_13030_MOESM1_ESM.pdf]

Applied Microbiology and Biotechnology

Engineering amino acid residues of pentacyclic triterpene synthases for improving the activity

Hao Guo[1†], Tongtong Chen[1†], Hanrong Zhu, Huiyan Wang, Yi-Xin Huo*

[1]Key Laboratory of Molecular Medicine and Biotherapy, School of Life Science, Beijing Institute of Technology, No. 5 South Zhongguancun Street, Beijing, 100081, PR China

†These authors contributed equally

Correspondence and requests for materials should be addressed to Y.-X.H. (Tel: 0086-68918158; email: huoyixin@bit.edu.cn)

This file includes:

Figure. S1 to S6

Table S1 to S3

References (1 to 2)

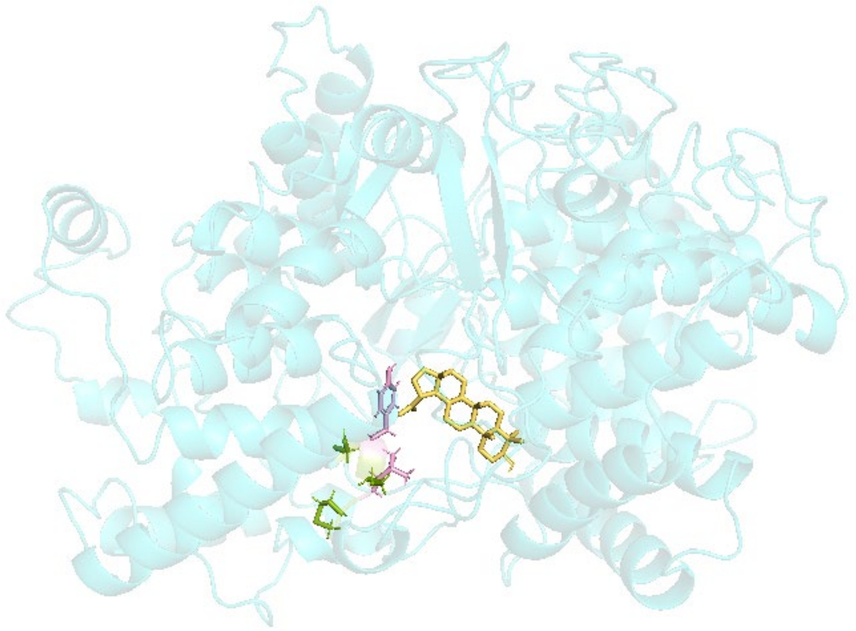

Figure S1 Molecular docking model of OEW and lupeol using PyMOL software.

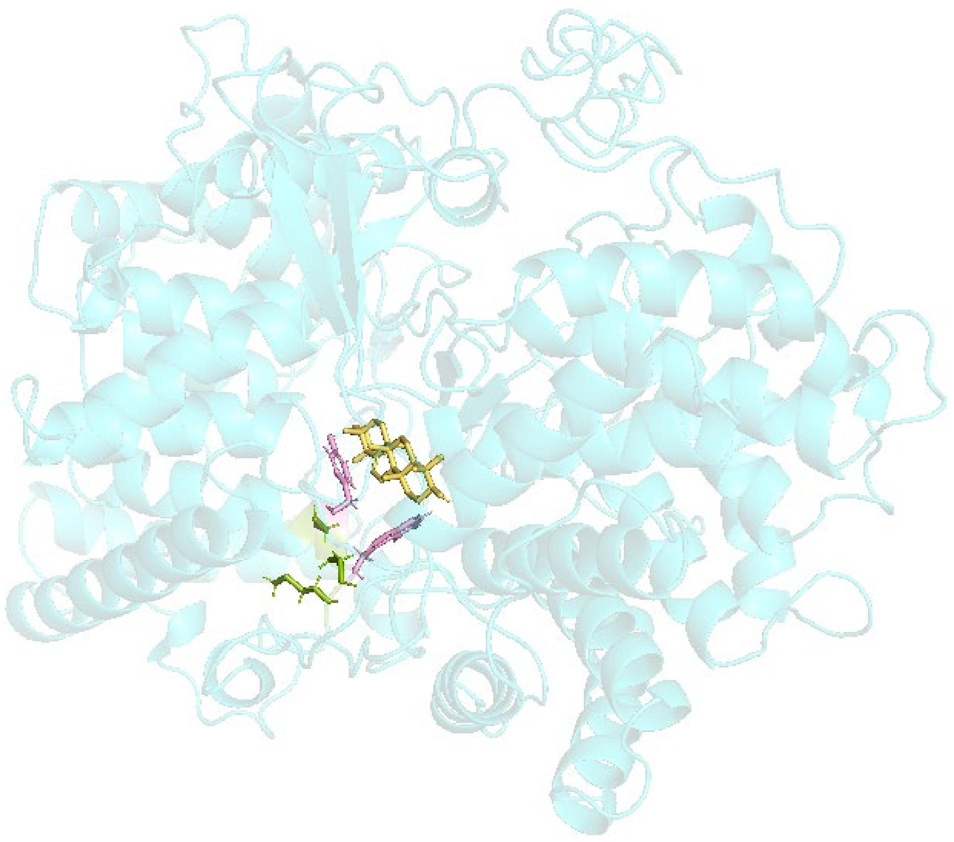

Figure S2 Molecular docking model of GgbAS and β-amyrin using PyMOL software.

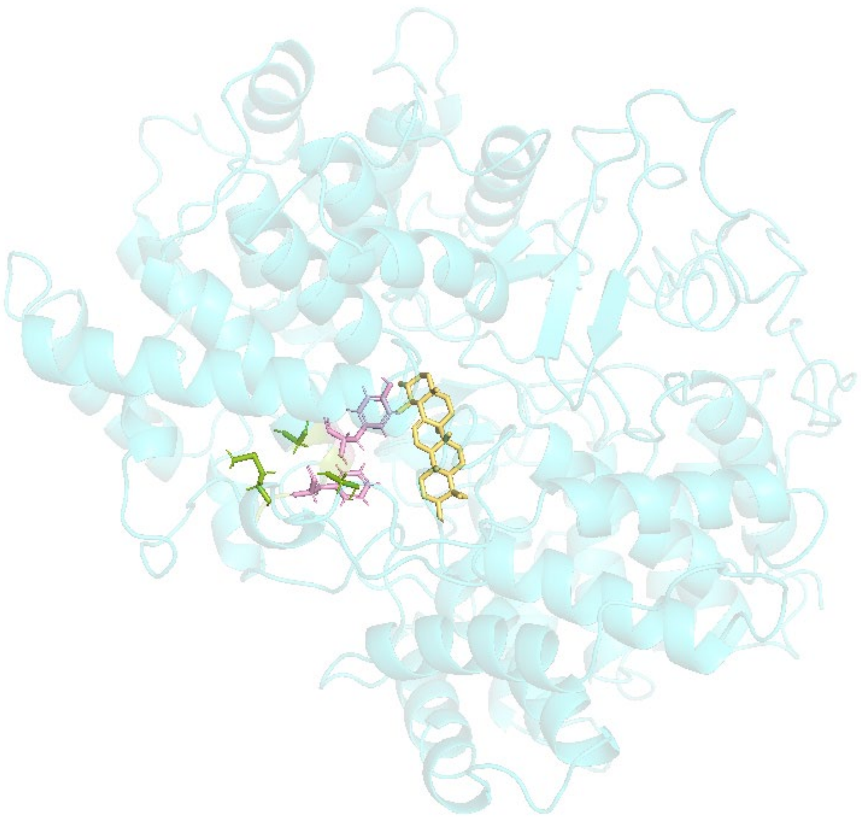

Figure S3 Molecular docking model of MdOSC1 and α-amyrin using PyMOL software.

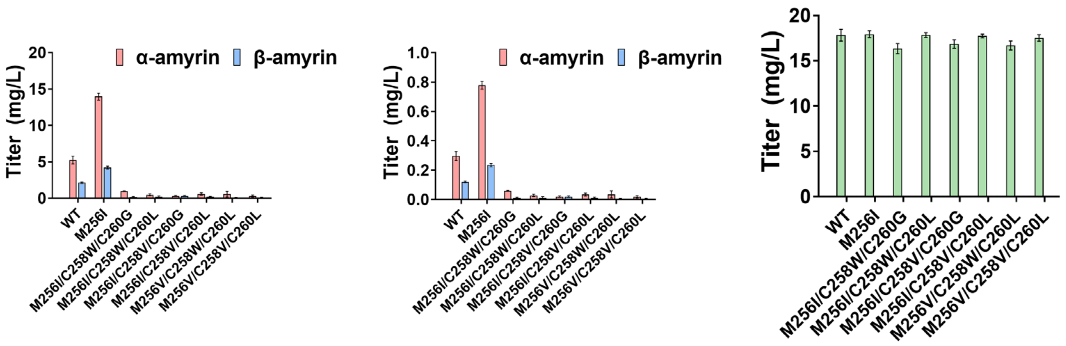

Figure S4 Influence of triple mutants on the activity of α-amyrin synthase from *Malus*×*domestica* (MdOSC1) on its activity.

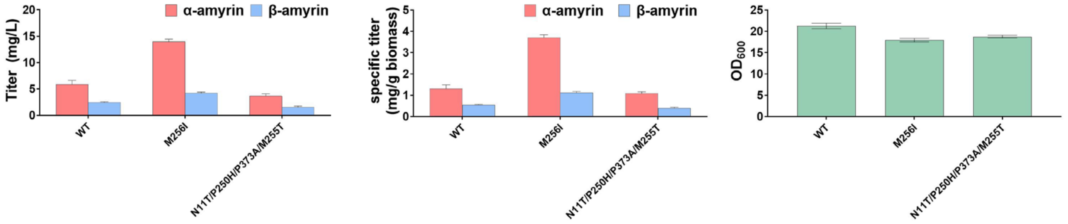

Figure S5 Influence of combinatorial mutagenesis of α-amyrin synthase from *Malus×domestica* (MdOSC1) on its activity.

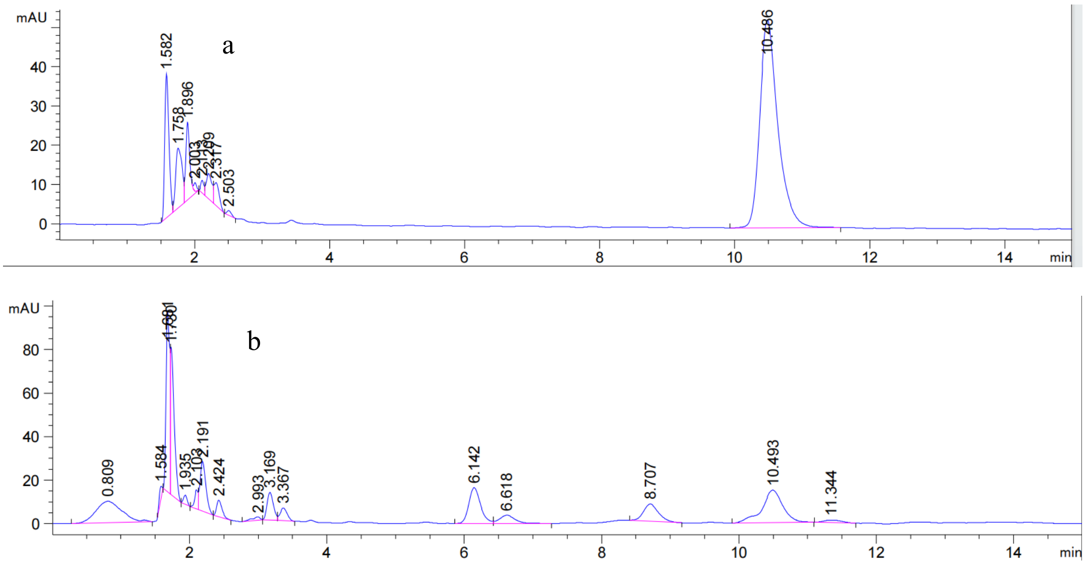

Figure S6 Representative chromatograms of (a) standard (b) sample.

Table S1 Plasmids used in this study

Plasmids and strains	Description	Source
Plasmids		
pCfB255	Amp^R, pBR322 origin, P_{PGK1}, P_{TEF1}, T_{ADH1}, T_{CYC1}, URA3	(Jensen et al., 2014)
p056-LOXP-GgbAS1-URA	pCfB255 derivative, P_{PGK1}-*GgbAS1*-T_{ADH1}, P_{TEF1}, T_{CYC1}, URA3	This study
p056-LOXP-GgbAS1^{M256A}-URA	pCfB255 derivative, P_{PGK1}-*GgbAS*^{M256A}-T_{ADH1}, P_{TEF1}, T_{CYC1}, URA3	This study
p056-LOXP-GgbAS^{M256G}-URA	pCfB255 derivative, P_{PGK1}-*GgbAS*^{M256G}-T_{ADH1}, P_{TEF1}, T_{CYC1}, URA3	This study
p056-LOXP-GgbAS^{M256I}-URA	pCfB255 derivative, P_{PGK1}-*GgbAS*^{M256I}-T_{ADH1}, P_{TEF1}, T_{CYC1}, URA3	This study
p056-LOXP-GgbAS^{M256L}-URA	pCfB255 derivative, P_{PGK1}-*GgbAS*^{M256L}-T_{ADH1}, P_{TEF1}, T_{CYC1}, URA3	This study

p056-LOXP-GgbAS^{M256P}-URA	pCfB255 derivative, P_{PGK1}-*GgbAS*^{M256P}-T_{ADH1}, P_{TEF1}, T_{CYC1}, URA3	This study
p056-LOXP-GgbAS^{M256V}-URA	pCfB255 derivative, P_{PGK1}-*GgbAS*^{M256V}-T_{ADH1}, P_{TEF1}, T_{CYC1}, URA3	This study
p056-LOXP-*GgbAS*^{M256F}-URA	pCfB255 derivative, P_{PGK1}-*GgbAS*^{M256F}-T_{ADH1}, P_{TEF1}, T_{CYC1}, URA3	This study
p056-LOXP-GgbAS^{M256W}-URA	pCfB255 derivative, P_{PGK1}-*GgbAS*^{M256W}-T_{ADH1}, P_{TEF1}, T_{CYC1}, URA3	This study
p056-LOXP-GgbAS^{M256Y}-URA	pCfB255 derivative, P_{PGK1}-*GgbAS*^{M256Y}-T_{ADH1}, P_{TEF1}, T_{CYC1}, URA3	This study
p056-LOXP-GgbAS^{M256D}-URA	pCfB255 derivative, P_{PGK1}-*GgbAS*^{M256D}-T_{ADH1}, P_{TEF1}, T_{CYC1}, URA3	This study
p056-LOXP-GgbAS^{M256E}-URA	pCfB255 derivative, P_{PGK1}-*GgbAS*^{M256E}-T_{ADH1}, P_{TEF1}, T_{CYC1}, URA3	This study
p056-LOXP-GgbAS^{M256R}-URA	pCfB255 derivative, P_{PGK1}-*GgbAS*^{M256R}-T_{ADH1}, P_{TEF1}, T_{CYC1}, URA3	This study
p056-LOXP-GgbAS^{M256H}-URA	pCfB255 derivative, P_{PGK1}-*GgbAS*^{M256H}-T_{ADH1}, P_{TEF1}, T_{CYC1}, URA3	This study
p056-LOXP-GgbAS^{M256K}-URA	pCfB255 derivative, P_{PGK1}-*GgbAS*^{M256K}-T_{ADH1}, P_{TEF1}, T_{CYC1}, URA3	This study
p056-LOXP-GgbAS^{M256S}-URA	pCfB255 derivative, P_{PGK1}-*GgbAS*^{M256S}-T_{ADH1}, P_{TEF1}, T_{CYC1}, URA3	This study
p056-LOXP-GgbAS^{M256T}-URA	pCfB255 derivative, P_{PGK1}-*GgbAS*^{M256T}-T_{ADH1}, P_{TEF1}, T_{CYC1}, URA3	This study
p056-LOXP-GgbAS^{M256C}-URA	pCfB255 derivative, P_{PGK1}-*GgbAS*^{M256C}-T_{ADH1}, P_{TEF1}, T_{CYC1}, URA3	This study
p056-LOXP-GgbAS^{M256N} -URA	pCfB255 derivative, P_{PGK1}-*GgbAS*^{M256N}-T_{ADH1}, P_{TEF1}, T_{CYC1}, URA3	This study
p056-LOXP-GgbAS^{M256Q}-URA	pCfB255 derivative, P_{PGK1}-*GgbAS*^{M256Q}-T_{ADH1}, P_{TEF1}, T_{CYC1}, URA3	This study
p056-LOXP-GgbAS^{C258A}-URA	pCfB255 derivative, P_{PGK1}-*GgbAS*^{C258A}-T_{ADH1}, P_{TEF1}, T_{CYC1}, URA3	This study
p056-LOXP-GgbAS^{C258G}-URA	pCfB255 derivative, P_{PGK1}-*GgbAS*^{C258G}-T_{ADH1}, P_{TEF1}, T_{CYC1}, URA3	This study
p056-LOXP-GgbAS^{C258I}-URA	PCfB255 derivative, P_{PGK1}-*GgbAS*^{C258I}-T_{ADH1}, P_{TEF1}, T_{CYC1}, URA3	This study
p056-LOXP-GgbAS^{C258L}-URA	pCfB255 derivative, P_{PGK1}-*GgbAS*^{C258L}-T_{ADH1}, P_{TEF1}, T_{CYC1}, URA3	This study

p056-LOXP-GgbAS^{C258P}-URA	pCfB255 derivative, P_{PGK1}-$GgbAS^{C258P}$-T_{ADH1}, P_{TEF1}, T_{CYC1}, URA3	This study
p056-LOXP-GgbAS^{C258V}-URA	pCfB255 derivative, P_{PGK1}-$GgbAS^{C258V}$-T_{ADH1}, P_{TEF1}, T_{CYC1}, URA3	This study
p056-LOXP-*GgbAS*^{C258F}-URA	pCfB255 derivative, P_{PGK1}-$GgbAS^{C258F}$-T_{ADH1}, P_{TEF1}, T_{CYC1}, URA3	This study
p056-LOXP-*GgbAS*^{C258W}-URA	pCfB255 derivative, P_{PGK1}-$GgbAS^{C258W}$-T_{ADH1}, P_{TEF1}, T_{CYC1}, URA3	This study
p056-LOXP-*GgbAS*^{C258Y}-URA	pCfB255 derivative, P_{PGK1}-$GgbAS^{C258Y}$-T_{ADH1}, P_{TEF1}, T_{CYC1}, URA3	This study
p056-LOXP-*GgbAS*^{C258D}-URA	pCfB255 derivative, P_{PGK1}-$GgbAS^{C258D}$-T_{ADH1}, P_{TEF1}, T_{CYC1}, URA3	This study
p056-LOXP-*GgbAS*^{C258E}-URA	pCfB255 derivative, P_{PGK1}-$GgbAS^{C258E}$-T_{ADH1}, P_{TEF1}, T_{CYC1}, URA3	This study
p056-LOXP-*GgbAS*^{C258R}-URA	pCfB255 derivative, P_{PGK1}-$GgbAS^{C258R}$-T_{ADH1}, P_{TEF1}, T_{CYC1}, URA3	This study
p056-LOXP-*GgbAS*^{C258H}-URA	pCfB255 derivative, P_{PGK1}-$GgbAS^{C258H}$-T_{ADH1}, P_{TEF1}, T_{CYC1}, URA3	This study
p056-LOXP-*GgbAS*^{C258K}-URA	pCfB255 derivative, P_{PGK1}-$GgbAS^{C258K}$-T_{ADH1}, P_{TEF1}, T_{CYC1}, URA3	This study
p056-LOXP-*GgbAS*^{C258S}-URA	pCfB255 derivative, P_{PGK1}-$GgbAS^{C258S}$-T_{ADH1}, P_{TEF1}, T_{CYC1}, URA3	This study
p056-LOXP-*GgbAS*^{C258T}-URA	pCfB255 derivative, P_{PGK1}-$GgbAS^{C258T}$-T_{ADH1}, P_{TEF1}, T_{CYC1}, URA3	This study
p056-LOXP-*GgbAS*^{C258M}-URA	pCfB255 derivative, P_{PGK1}-$GgbAS^{C258M}$-T_{ADH1}, P_{TEF1}, T_{CYC1}, URA3	This study
p056-LOXP-*GgbAS*^{C258N}-URA	pCfB255 derivative, P_{PGK1}-$GgbAS^{C258N}$-T_{ADH1}, P_{TEF1}, T_{CYC1}, URA3	This study
p056-LOXP-*GgbAS*^{C258Q}-URA	pCfB255 derivative, P_{PGK1}-$GgbAS^{C258Q}$-T_{ADH1}, P_{TEF1}, T_{CYC1}, URA3	This study
p056-LOXP-*GgbAS*^{C260A} -URA	pCfB255 derivative, P_{PGK1}-$GgbAS^{C260A}$-T_{ADH1}, P_{TEF1}, T_{CYC1}, URA3	This study
p056-LOXP-*GgbAS*^{C260G}-URA	pCfB255 derivative, P_{PGK1}-$GgbAS^{C260G}$-T_{ADH1}, P_{TEF1}, T_{CYC1}, URA3	This study
p056-LOXP-*GgbAS*^{C260I}-URA	pCfB255 derivative, P_{PGK1}-$GgbAS^{C260I}$-T_{ADH1}, P_{TEF1}, T_{CYC1}, URA3	This study
p056-LOXP-*GgbAS*^{C260L} -URA	pCfB255 derivative, P_{PGK1}-$GgbAS^{C260L}$-T_{ADH1}, P_{TEF1}, T_{CYC1}, URA3	This study

p056-LOXP-$GgbAS^{C260P}$-URA	pCfB255 derivative, P_{PGK1}-$GgbAS^{C260P}$-T_{ADH1}, P_{TEF1}, T_{CYC1}, URA3	This study
p056-LOXP-$GgbAS^{C260V}$-URA	pCfB255 derivative, P_{PGK1}-$GgbAS^{C260V}$-T_{ADH1}, P_{TEF1}, T_{CYC1}, URA3	This study
p056-LOXP-$GgbAS^{C260F}$-URA	pCfB255 derivative, P_{PGK1}-$GgbAS^{C260F}$-T_{ADH1}, P_{TEF1}, T_{CYC1}, URA3	This study
p056-LOXP-$GgbAS^{C260W}$-URA	pCfB255 derivative, P_{PGK1}-$GgbAS^{C260W}$-T_{ADH1}, P_{TEF1}, T_{CYC1}, URA3	This study
p056-LOXP-$GgbAS^{C260Y}$-URA	pCfB255 derivative, P_{PGK1}-$GgbAS^{C260Y}$-T_{ADH1}, P_{TEF1}, T_{CYC1}, URA3	This study
p056-LOXP-$GgbAS^{C260D}$-URA	pCfB255 derivative, P_{PGK1}-$GgbAS^{C260D}$-T_{ADH1}, P_{TEF1}, T_{CYC1}, URA3	This study
p056-LOXP-$GgbAS^{C260E}$-URA	pCfB255 derivative, P_{PGK1}-$GgbAS^{C260E}$-T_{ADH1}, P_{TEF1}, T_{CYC1}, URA3	This study
p056-LOXP-$GgbAS^{C260R}$-URA	pCfB255 derivative, P_{PGK1}-$GgbAS^{C260R}$-T_{ADH1}, P_{TEF1}, T_{CYC1}, URA3	This study
p056-LOXP-$GgbAS^{C260H}$-URA	pCfB255 derivative, P_{PGK1}-$GgbAS^{C260H}$-T_{ADH1}, P_{TEF1}, T_{CYC1}, URA3	This study
p056-LOXP-$GgbAS^{C260K}$-URA	pCfB255 derivative, P_{PGK1}-$GgbAS^{C260K}$-T_{ADH1}, P_{TEF1}, T_{CYC1}, URA3	This study
p056-LOXP-$GgbAS^{C260S}$-URA	pCfB255 derivative, P_{PGK1}-$GgbAS^{C260S}$-T_{ADH1}, P_{TEF1}, T_{CYC1}, URA3	This study
p056-LOXP-$GgbAS^{C260T}$-URA	pCfB255 derivative P_{PGK1}-$GgbAS^{C260T}$-T_{ADH1}, P_{TEF1}, T_{CYC1}, URA3	This study
p056-LOXP-$GgbAS^{C260M}$ -URA	pCfB255 derivative, P_{PGK1}-$GgbAS^{C260M}$-T_{ADH1}, P_{TEF1}, T_{CYC1}, URA3	This study
p056-LOXP-$GgbAS^{C260N}$-URA	pCfB255 derivative, P_{PGK1}-$GgbAS^{C260N}$-T_{ADH1}, P_{TEF1}, T_{CYC1}, URA3	This study
p056-LOXP-$GgbAS^{C260Q}$-URA	pCfB255 derivative, P_{PGK1}-$GgbAS^{C260Q}$-T_{ADH1}, P_{TEF1}, T_{CYC1}, URA3	This study
p056-LOXP-$GgbAS^{M256Q/C258A}$-URA	pCfB255 derivative, P_{PGK1}-$GgbAS^{M256Q/C258A}$-T_{ADH1}, P_{TEF1}, T_{CYC1}, URA3	This study
p056-LOXP-$GgbAS^{M256Q/C258V}$-URA	pCfB255 derivative, P_{PGK1}-$GgbAS^{M256Q/C258V}$-T_{ADH1}, P_{TEF1}, T_{CYC1}, URA3	This study
p056-LOXP-$GgbAS^{M256A/C258A}$-	pCfB255 derivative, P_{PGK1}-$GgbAS^{M256A/C258A}$-T_{ADH1}, P_{TEF1}, T_{CYC1}, URA3	This study

URA		
p056-LOXP-*GgbAS*$^{M256A/C258V}$-URA	pCfB255 derivative, P_{PGK1}-*GgbAS*$^{M256A/C258V}$-T_{ADH1}, P_{TEF1}, T_{CYC1}, URA3	This study
p056-LOXP-*GgbAS*$^{M256Q/C260W}$-URA	pCfB255 derivative, P_{PGK1}-*GgbAS*$^{M256Q/C260W}$-T_{ADH1}, P_{TEF1}, T_{CYC1}, URA3	This study
p056-LOXP-*GgbAS*$^{M256Q/C260H}$-URA	pCfB255 derivative, P_{PGK1}-*GgbAS*$^{M256Q/C260H}$-T_{ADH1}, P_{TEF1}, T_{CYC1}, URA3	This study
p056-LOXP-*GgbAS*$^{M256A/C260W}$-URA	pCfB255 derivative, P_{PGK1}-*GgbAS*$^{M256A/C260W}$-T_{ADH1}, P_{TEF1}, T_{CYC1}, URA3	This study
p056-LOXP-*GgbAS*$^{M256A/C260H}$-URA	pCfB255 derivative, P_{PGK1}-*GgbAS*$^{M256A/C258H}$-T_{ADH1}, P_{TEF1}, T_{CYC1}, URA3	This study
p056-LOXP-*GgbAS*$^{C258A/C260W}$-URA	pCfB255 derivative, P_{PGK1}-*GgbAS*$^{C258A/C260W}$-T_{ADH1}, P_{TEF1}, T_{CYC1}, URA3	This study
p056-LOXP-*GgbAS*$^{C258A/C260H}$-URA	pCfB255 derivative, P_{PGK1}-*GgbAS*$^{C258A/C260H}$-T_{ADH1}, P_{TEF1}, T_{CYC1}, URA3	This study
p056-LOXP-*GgbAS*$^{C258V/C260W}$-URA	pCfB255 derivative, P_{PGK1}-*GgbAS*$^{C258V/C260W}$-T_{ADH1}, P_{TEF1}, T_{CYC1}, URA3	This study
p056-LOXP-*GgbAS*$^{C258V/C260H}$-URA	pCfB255 derivative, P_{PGK1}-*GgbAS*$^{C258V/C260H}$-T_{ADH1}, P_{TEF1}, T_{CYC1}, URA3	This study
p056-LOXP-*GgbAS*$^{M256A/C258A/C260W}$-URA	pCfB255 derivative, P_{PGK1}-*GgbAS*$^{M256A/C258A/C260W}$-T_{ADH1}, P_{TEF1}, T_{CYC1}, URA3	This study
p056-LOXP-*GgbAS*$^{M256A/C258V/C260W}$-URA	pCfB255 derivative, P_{PGK1}-*GgbAS*$^{M256A/C258V/C260W}$-T_{ADH1}, P_{TEF1}, T_{CYC1}, URA3	This study
p056-LOXP-*GgbAS*$^{M256A/C258A/C260H}$-URA	pCfB255 derivative, P_{PGK1}-*GgbAS*$^{M256A/C258A/C260H}$-T_{ADH1}, P_{TEF1}, T_{CYC1}, URA3	This study

p056-LOXP-$GgbAS^{M256A/C258V/C260H}$-URA	pCfB255 derivative, P_{PGK1}-$GgbAS^{M256A/C258V/C260H}$-$T_{ADH1}$, P_{TEF1}, T_{CYC1}, URA3	This study
p056-LOXP-$GgbAS^{M256Q/C258A/C260W}$-URA	pCfB255 derivative, P_{PGK1}-$GgbAS^{M256Q/C258A/C260W}$-$T_{ADH1}$, P_{TEF1}, T_{CYC1}, URA3	This study
p056-LOXP-$GgbAS^{M256Q/C258V/C260W}$-URA	pCfB255 derivative, P_{PGK1}-$GgbAS^{M256Q/C258V/C260W}$-$T_{ADH1}$, P_{TEF1}, T_{CYC1}, URA3	This study
p056-LOXP-$GgbAS^{M256Q/C258A/C260H}$-URA	pCfB255 derivative, P_{PGK1}-$GgbAS^{M256Q/C258A/C260H}$-$T_{ADH1}$, P_{TEF1}, T_{CYC1}, URA3	This study
p056-LOXP-$GgbAS^{M256Q/C258V/C260H}$-URA	pCfB255 derivative, P_{PGK1}-$GgbAS^{M256Q/C258V/C260H}$-$T_{ADH1}$, P_{TEF1}, T_{CYC1}, URA3	This study
p056-LOXP-OEW-URA	pCfB255 derivative, P_{PGK1}-OEW-T_{ADH1}, P_{TEF1}, T_{CYC1}, URA3	This study
p056-LOXP-OEW^{M256A}-URA	pCfB255 derivative, P_{PGK1}-OEW^{M256A}-T_{ADH1}, P_{TEF1}, T_{CYC1}, URA3	This study
p056-LOXP-OEW^{M256G}-URA	pCfB255 derivative, P_{PGK1}-OEW^{M256G}-T_{ADH1}, P_{TEF1}, T_{CYC1}, URA3	This study
p056-LOXP-OEW^{M256I}-URA	pCfB255 derivative, P_{PGK1}-OEW^{M256I}-T_{ADH1}, P_{TEF1}, T_{CYC1}, URA3	This study
p056-LOXP-OEW^{M256L}-URA	pCfB255 derivative, P_{PGK1}-OEW^{M256L}-T_{ADH1}, P_{TEF1}, T_{CYC1}, URA3	This study
p056-LOXP-OEW^{M256P}-URA	pCfB255 derivative, P_{PGK1}-OEW^{M256P}-T_{ADH1}, P_{TEF1}, T_{CYC1}, URA3	This study
p056-LOXP-OEW^{M256V}-URA	pCfB255 derivative, P_{PGK1}-OEW^{M256V}-T_{ADH1}, P_{TEF1}, T_{CYC1}, URA3	This study
p056-LOXP-OEW^{M256F} -URA	pCfB255 derivative, P_{PGK1}-OEW^{M256F}-T_{ADH1}, P_{TEF1}, T_{CYC1}, URA3	This study
p056-LOXP-OEW^{M256W}-URA	pCfB255 derivative, P_{PGK1}-OEW^{M256W}-T_{ADH1}, P_{TEF1}, T_{CYC1}, URA3	This study
p056-LOXP-OEW^{M256Y}-URA	pCfB255 derivative, P_{PGK1}-OEW^{M256Y}-T_{ADH1}, P_{TEF1}, T_{CYC1}, URA3	This study
p056-LOXP-OEW^{M256D}-URA	pCfB255 derivative, P_{PGK1}-OEW^{M256D}-T_{ADH1}, P_{TEF1}, T_{CYC1}, URA3	This study

p056-LOXP-OEW^{M256E}-URA	pCfB255 derivative, P_{PGK1}-OEW^{M256E}-T_{ADH1}, P_{TEF1}, T_{CYC1}, URA3	This study
p056-LOXP-OEW^{M256R}-URA	pCfB255 derivative, P_{PGK1}-OEW^{M256R}-T_{ADH1}, P_{TEF1}, T_{CYC1}, URA3	This study
p056-LOXP-OEW^{M256H}-URA	pCfB255 derivative, P_{PGK1}-OEW^{M256H}-T_{ADH1}, P_{TEF1}, T_{CYC1}, URA3	This study
p056-LOXP-OEW^{M256K}-URA	pCfB255 derivative, P_{PGK1}-OEW^{M256K}-T_{ADH1}, P_{TEF1}, T_{CYC1}, URA3	This study
p056-LOXP-OEW^{M256S}-URA	pCfB255 derivative, P_{PGK1}-OEW^{M256S}-T_{ADH1}, P_{TEF1}, T_{CYC1}, URA3	This study
p056-LOXP-OEW^{M256T}-URA	pCfB255 derivative, P_{PGK1}-OEW^{M256T}-T_{ADH1}, P_{TEF1}, T_{CYC1}, URA3	This study
p056-LOXP-OEW^{M256C}-URA	pCfB255 derivative, P_{PGK1}-OEW^{M256C}-T_{ADH1}, P_{TEF1}, T_{CYC1}, URA3	This study
p056-LOXP-OEW^{M256N}-URA	pCfB255 derivative, P_{PGK1}-OEW^{M256N}-T_{ADH1}, P_{TEF1}, T_{CYC1}, URA3	This study
p056-LOXP-OEW^{M256Q}-URA	pCfB255 derivative, P_{PGK1}-OEW^{M256Q}-T_{ADH1}, P_{TEF1}, T_{CYC1}, URA3	This study
p056-LOXP-OEW^{C258A}-URA	pCfB255 derivative, P_{PGK1}-OEW^{C258A}-T_{ADH1}, P_{TEF1}, T_{CYC1}, URA3	This study
p056-LOXP-OEW^{C258G}-URA	pCfB255 derivative, P_{PGK1}-OEW^{C258G}-T_{ADH1}, P_{TEF1}, T_{CYC1}, URA3	This study
p056-LOXP-OEW^{C258I}-URA	pCfB255 derivative, P_{PGK1}-OEW^{C258I}-T_{ADH1}, P_{TEF1}, T_{CYC1}, URA3	This study
p056-LOXP-OEW^{C258L}-URA	pCfB255 derivative, P_{PGK1}-OEW^{C258L}-T_{ADH1}, P_{TEF1}, T_{CYC1}, URA3	This study
p056-LOXP-OEW^{C258P}-URA	pCfB255 derivative, P_{PGK1}-OEW^{C258P}-T_{ADH1}, P_{TEF1}, T_{CYC1}, URA3	This study
p056-LOXP-OEW^{C258V}-URA	pCfB255 derivative, P_{PGK1}-OEW^{C258V}-T_{ADH1}, P_{TEF1}, T_{CYC1}, URA3	This study
p056-LOXP-OEW^{C258F}-URA	pCfB255 derivative, P_{PGK1}-OEW^{C258F}-T_{ADH1}, P_{TEF1}, T_{CYC1}, URA3	This study
p056-LOXP-OEW^{C258W}-URA	pCfB255 derivative, P_{PGK1}-OEW^{C258W}-T_{ADH1}, P_{TEF1}, T_{CYC1}, URA3	This study
p056-LOXP-OEW^{C258Y}-URA	pCfB255 derivative, P_{PGK1}-OEW^{C258Y}-T_{ADH1}, P_{TEF1}, T_{CYC1}, URA3	This study
p056-LOXP-OEW^{C258D}-URA	pCfB255 derivative, P_{PGK1}-OEW^{C258D}-T_{ADH1}, P_{TEF1}, T_{CYC1}, URA3	This study

p056-LOXP-OEW^{C258E}-URA	pCfB255 derivative, P_{PGK1}-OEW^{C258E}-T_{ADH1}, P_{TEF1}, T_{CYC1}, URA3	This study
p056-LOXP-OEW^{C258R}-URA	pCfB255 derivative, P_{PGK1}-OEW^{C258R}-T_{ADH1}, P_{TEF1}, T_{CYC1}, URA3	This study
p056-LOXP-OEW^{C258H}-URA	pCfB255 derivative, P_{PGK1}-OEW^{C258H}-T_{ADH1}, P_{TEF1}, T_{CYC1}, URA3	This study
p056-LOXP-OEW^{C258K}-URA	pCfB255 derivative, P_{PGK1}-OEW^{C258K}-T_{ADH1}, P_{TEF1}, T_{CYC1}, URA3	This study
p056-LOXP-OEW^{C258S}-URA	pCfB255 derivative, P_{PGK1}-OEW^{C258S}-T_{ADH1}, P_{TEF1}, T_{CYC1}, URA3	This study
p056-LOXP-OEW^{C258T}-URA	pCfB255 derivative, P_{PGK1}-OEW^{C258T}-T_{ADH1}, P_{TEF1}, T_{CYC1}, URA3	This study
p056-LOXP-OEW^{C258M}-URA	pCfB255 derivative, P_{PGK1}-OEW^{C258M}-T_{ADH1}, P_{TEF1}, T_{CYC1}, URA3	This study
p056-LOXP-OEW^{C258N}-URA	pCfB255 derivative, P_{PGK1}-OEW^{C258N}-T_{ADH1}, P_{TEF1}, T_{CYC1}, URA3	This study
p056-LOXP-OEW^{C258Q}-URA	pCfB255 derivative, P_{PGK1}-OEW^{C258Q}-T_{ADH1}, P_{TEF1}, T_{CYC1}, URA3	This study
p056-LOXP-OEW^{C260A}-URA	pCfB255 derivative, P_{PGK1}-OEW^{C260A}-T_{ADH1}, P_{TEF1}, T_{CYC1}, URA3	This study
p056-LOXP-OEW^{C260G}-URA	pCfB255 derivative, P_{PGK1}-OEW^{C260G}-T_{ADH1}, P_{TEF1}, T_{CYC1}, URA3	This study
p056-LOXP-OEW^{C260I}-URA	pCfB255 derivative, P_{PGK1}-OEW^{C260I}-T_{ADH1}, P_{TEF1}, T_{CYC1}, URA3	This study
p056-LOXP-OEW^{C260L}-URA	pCfB255 derivative, P_{PGK1}-OEW^{C260L}-T_{ADH1}, P_{TEF1}, T_{CYC1}, URA3	This study
p056-LOXP-OEW^{C260P}-URA	pCfB255 derivative, P_{PGK1}-OEW^{C260P}-T_{ADH1}, P_{TEF1}, T_{CYC1}, URA3	This study
p056-LOXP-OEW^{C260V}-URA	pCfB255 derivative, P_{PGK1}-OEW^{C260V}-T_{ADH1}, P_{TEF1}, T_{CYC1}, URA3	This study
p056-LOXP-OEW^{C260F}-URA	pCfB255 derivative, P_{PGK1}-OEW^{C260F}-T_{ADH1}, P_{TEF1}, T_{CYC1}, URA3	This study
p056-LOXP-OEW^{C260W}-URA	pCfB255 derivative, P_{PGK1}-OEW^{C260W}-T_{ADH1}, P_{TEF1}, T_{CYC1}, URA3	This study
p056-LOXP-OEW^{C260Y}-URA	pCfB255 derivative, P_{PGK1}-OEW^{C260Y}-T_{ADH1}, P_{TEF1}, T_{CYC1}, URA3	This study
p056-LOXP-OEW^{C260D}-URA	pCfB255 derivative, P_{PGK1}-OEW^{C260D}-T_{ADH1}, P_{TEF1}, T_{CYC1}, URA3	This study

p056-LOXP-OEW^{C260E}-URA	pCfB255 derivative, P_{PGK1}-OEW^{C260E}-T_{ADH1}, P_{TEF1}, T_{CYC1}, URA3	This study
p056-LOXP-OEW^{C260R}-URA	pCfB255 derivative, P_{PGK1}-OEW^{C260R}-T_{ADH1}, P_{TEF1}, T_{CYC1}, URA3	This study
p056-LOXP-OEW^{C260H}-URA	pCfB255 derivative, P_{PGK1}-OEW^{C260H}-T_{ADH1}, P_{TEF1}, T_{CYC1}, URA3	This study
p056-LOXP-OEW^{C260K}-URA	pCfB255 derivative, P_{PGK1}-OEW^{C260K}-T_{ADH1}, P_{TEF1}, T_{CYC1}, URA3	This study
p056-LOXP-OEW^{C260S}-URA	Amp^{R}, pBR322 origin, P_{PGK1}-OEW^{C260S}-T_{ADH1}, P_{TEF1}, T_{CYC1}, URA3	This study
p056-LOXP-OEW^{C260T}-URA	Amp^{R}, pBR322 origin, P_{PGK1}-OEW^{C260T}-T_{ADH1}, P_{TEF1}, T_{CYC1}, URA3	This study
p056-LOXP-OEW^{C260M}-URA	Amp^{R}, pBR322 origin, P_{PGK1}-OEW^{C260M}-T_{ADH1}, P_{TEF1}, T_{CYC1}, URA3	This study
p056-LOXP-OEW^{C260N}-URA	Amp^{R}, pBR322 origin, P_{PGK1}-OEW^{C260N}-T_{ADH1}, P_{TEF1}, T_{CYC1}, URA3	This study
p056-LOXP-OEW^{C260Q}-URA	Amp^{R}, pBR322 origin, P_{PGK1}-OEW^{C260Q}-T_{ADH1}, P_{TEF1}, T_{CYC1}, URA3	This study
p056-LOXP-OEW$^{M256V/C260A}$-URA	Amp^{R}, pBR322 origin, P_{PGK1}-$OEW^{M256V/C260A}$-T_{ADH1}, P_{TEF1}, T_{CYC1}, URA3	This study
p056-LOXP-OEW$^{M256L/C260A}$-URA	Amp^{R}, pBR322 origin, P_{PGK1}-$OEW^{M256L/C260A}$-T_{ADH1}, P_{TEF1}, T_{CYC1}, URA3	This study
p056-LOXP-OEW$^{M256C/C260A}$-URA	Amp^{R}, pBR322 origin, P_{PGK1}-$OEW^{M256C/C260A}$-T_{ADH1}, P_{TEF1}, T_{CYC1}, URA3	This study
p056-LOXP-OEW$^{M256I/C260A}$-URA	Amp^{R}, pBR322 origin, P_{PGK1}-$OEW^{M256I/C260A}$-T_{ADH1}, P_{TEF1}, T_{CYC1}, URA3	This study
p056-LOXP-OEW$^{M256Y/C260A}$-URA	Amp^{R}, pBR322 origin, P_{PGK1}-$OEW^{M256Y/C260A}$-T_{ADH1}, P_{TEF1}, T_{CYC1}, URA3	This study
p056-LOXP-OEW$^{M256V/C260G}$-URA	Amp^{R}, pBR322 origin, P_{PGK1}-$OEW^{M256V/C260G}$-T_{ADH1}, P_{TEF1}, T_{CYC1}, URA3	This study
p056-LOXP-OEW$^{M256L/C260G}$-URA	Amp^{R}, pBR322 origin, P_{PGK1}-$OEW^{M256L/C260G}$-T_{ADH1}, P_{TEF1}, T_{CYC1}, URA3	This study
p056-LOXP-OEW$^{M256C/C260G}$-URA	Amp^{R}, pBR322 origin, P_{PGK1}-OEW$^{M256C/C260G}$-T_{ADH1}, P_{TEF1}, T_{CYC1}, URA3	This study
p056-LOXP-OEW$^{M256I/C260G}$-URA	Amp^{R}, pBR322 origin, P_{PGK1}-$OEW^{M256I/C260G}$-T_{ADH1}, P_{TEF1}, T_{CYC1}, URA3	This study
p056-LOXP-OEW$^{M256Y/C260G}$-URA	Amp^{R}, pBR322 origin, P_{PGK1}-$OEW^{M256Y/C260G}$-T_{ADH1}, P_{TEF1}, T_{CYC1}, URA3	This study

p056-LOXP-MdOSC1-URA	Amp^{R}, pBR322 origin, P_{PGK1}-*MdOSC1*-T_{ADH1}, P_{TEF1}, T_{CYC1}, URA3	This study
p056-LOXP-MdOSC1^{M255A}-URA	Amp^{R}, pBR322 origin, P_{PGK1}-*MdOSC1*^{M255A}-T_{ADH1}, P_{TEF1}, T_{CYC1}, URA3	This study
p056-LOXP-MdOSC1^{M255G}-URA	Amp^{R}, pBR322 origin, P_{PGK1}-*MdOSC1*^{M255G}-T_{ADH1}, P_{TEF1}, T_{CYC1}, URA3	This study
p056-LOXP-MdOSC1^{M255I}-URA	Amp^{R}, pBR322 origin, P_{PGK1}-*MdOSC1*^{M255I}-T_{ADH1}, P_{TEF1}, T_{CYC1}, URA3	This study
p056-LOXP-MdOSC1^{M255L}-URA	pCfB255 derivative, P_{PGK1}-*MdOSC1*^{M255L}-T_{ADH1}, P_{TEF1}, T_{CYC1}, URA3	This study
p056-LOXP-MdOSC1^{M255P}-URA	pCfB255 derivative, P_{PGK1}-*MdOSC1*^{M255P}-T_{ADH1}, P_{TEF1}, T_{CYC1}, URA3	This study
p056-LOXP-MdOSC1^{M255V}-URA	pCfB255 derivative, P_{PGK1}-*MdOSC1*^{M255V}-T_{ADH1}, P_{TEF1}, T_{CYC1}, URA3	This study
p056-LOXP-MdOSC1^{M255F}-URA	pCfB255 derivative, P_{PGK1}-*MdOSC1*^{M255F}-T_{ADH1}, P_{TEF1}, T_{CYC1}, URA3	This study
p056-LOXP-MdOSC1^{M255W}-URA	pCfB255 derivative, P_{PGK1}-*MdOSC1*^{M255W}-T_{ADH1}, P_{TEF1}, T_{CYC1}, URA3	This study
p056-LOXP-MdOSC1^{M255Y}-URA	pCfB255 derivative, P_{PGK1}-*MdOSC1*^{M255Y}-T_{ADH1}, P_{TEF1}, T_{CYC1}, URA3	This study
p056-LOXP-MdOSC1^{M255D}-URA	pCfB255 derivative, P_{PGK1}-*MdOSC1*^{M255A}-T_{ADH1}, P_{TEF1}, T_{CYC1}, URA3	This study
p056-LOXP-MdOSC1^{M255E}-URA	pCfB255 derivative, P_{PGK1}-*MdOSC1*^{M255E}-T_{ADH1}, P_{TEF1}, T_{CYC1}, URA3	This study
p056-LOXP-MdOSC1^{M255R}-URA	pCfB255 derivative, P_{PGK1}-*MdOSC1*^{M255R}-T_{ADH1}, P_{TEF1}, T_{CYC1}, URA3	This study
p056-LOXP-MdOSC1^{M255H}-URA	pCfB255 derivative, P_{PGK1}-*MdOSC1*^{M255H}-T_{ADH1}, P_{TEF1}, T_{CYC1}, URA3	This study
p056-LOXP-MdOSC1^{M255K}-URA	pCfB255 derivative, P_{PGK1}-*MdOSC1*^{M255A}-T_{ADH1}, P_{TEF1}, T_{CYC1}, URA3	This study
p056-LOXP-MdOSC1^{M255S}-URA	pCfB255 derivative, P_{PGK1}-*MdOSC1*^{M255S}-T_{ADH1}, P_{TEF1}, T_{CYC1}, URA3	This study
p056-LOXP-MdOSC1^{M255T}-URA	pCfB255 derivative, P_{PGK1}-*MdOSC1*^{M255T}-T_{ADH1}, P_{TEF1}, T_{CYC1}, URA3	This study
p056-LOXP-MdOSC1^{M255C}-URA	pCfB255 derivative, P_{PGK1}-*MdOSC1*^{M255C}-T_{ADH1}, P_{TEF1}, T_{CYC1}, URA3	This study
p056-LOXP-MdOSC1^{M255N}-URA	pCfB255 derivative, P_{PGK1}-*MdOSC1*^{M255N}-T_{ADH1}, P_{TEF1}, T_{CYC1}, URA3	This study

p056-LOXP-MdOSC1^{M255Q}-URA	pCfB255 derivative, P_{PGK1}-$MdOSC1^{M255Q}$-T_{ADH1}, P_{TEF1}, T_{CYC1}, URA3	This study
p056-LOXP-MdOSC1^{C257A}-URA	pCfB255 derivative, P_{PGK1}-$MdOSC1^{C257A}$-T_{ADH1}, P_{TEF1}, T_{CYC1}, URA3	This study
p056-LOXP-MdOSC1^{C257G}-URA	pCfB255 derivative, P_{PGK1}-$MdOSC1^{C257G}$-T_{ADH1}, P_{TEF1}, T_{CYC1}, URA3	This study
p056-LOXP-MdOSC1^{C257I}-URA	pCfB255 derivative, P_{PGK1}-$MdOSC1^{C257I}$-T_{ADH1}, P_{TEF1}, T_{CYC1}, URA3	This study
p056-LOXP-MdOSC1^{C257L}-URA	pCfB255 derivative, P_{PGK1}-$MdOSC1^{C257L}$-T_{ADH1}, P_{TEF1}, T_{CYC1}, URA3	This study
p056-LOXP-MdOSC1^{C257P}-URA	pCfB255 derivative, P_{PGK1}-$MdOSC1^{C257P}$-T_{ADH1}, P_{TEF1}, T_{CYC1}, URA3	This study
p056-LOXP-MdOSC1^{C257V}-URA	pCfB255 derivative, P_{PGK1}-$MdOSC1^{C257V}$-T_{ADH1}, P_{TEF1}, T_{CYC1}, URA3	This study
p056-LOXP-MdOSC1^{C257F}-URA	pCfB255 derivative, P_{PGK1}-$MdOSC1^{C257F}$-T_{ADH1}, P_{TEF1}, T_{CYC1}, URA3	This study
p056-LOXP-MdOSC1^{C257W}-URA	pCfB255 derivative, P_{PGK1}-$MdOSC1^{C257W}$-T_{ADH1}, P_{TEF1}, T_{CYC1}, URA3	This study
p056-LOXP-MdOSC1^{C257Y}-URA	pCfB255 derivative, P_{PGK1}-$MdOSC1^{C257Y}$-T_{ADH1}, P_{TEF1}, T_{CYC1}, URA3	This study
p056-LOXP-MdOSC1^{C257D}-URA	pCfB255 derivative, P_{PGK1}-$MdOSC1^{C257D}$-T_{ADH1}, P_{TEF1}, T_{CYC1}, URA3	This study
p056-LOXP-MdOSC1^{C257E}-URA	pCfB255 derivative, P_{PGK1}-$MdOSC1^{C257E}$-T_{ADH1}, P_{TEF1}, T_{CYC1}, URA3	This study
p056-LOXP-MdOSC1^{C257R}-URA	pCfB255 derivative, P_{PGK1}-$MdOSC1^{C257R}$-T_{ADH1}, P_{TEF1}, T_{CYC1}, URA3	This study
p056-LOXP-MdOSC1^{C257H}-URA	pCfB255 derivative, P_{PGK1}-$MdOSC1^{C257H}$-T_{ADH1}, P_{TEF1}, T_{CYC1}, URA3	This study
p056-LOXP-MdOSC1^{C257K}-URA	pCfB255 derivative, P_{PGK1}-$MdOSC1^{C257K}$-T_{ADH1}, P_{TEF1}, T_{CYC1}, URA3	This study
p056-LOXP-MdOSC1^{C257S}-URA	pCfB255 derivative, P_{PGK1}-$MdOSC1^{C257S}$-T_{ADH1}, P_{TEF1}, T_{CYC1}, URA3	This study
p056-LOXP-MdOSC1^{C257T}-URA	pCfB255 derivative, P_{PGK1}-$MdOSC1^{C257T}$-T_{ADH1}, P_{TEF1}, T_{CYC1}, URA3	This study
p056-LOXP-MdOSC1^{C257M}-URA	pCfB255 derivative, P_{PGK1}-$MdOSC1^{C257M}$-T_{ADH1}, P_{TEF1}, T_{CYC1}, URA3	This study
p056-LOXP-MdOSC1^{C257N}-URA	pCfB255 derivative, P_{PGK1}-$MdOSC1^{C257N}$-T_{ADH1}, P_{TEF1}, T_{CYC1}, URA3	This study

p056-LOXP-MdOSC1^{C257Q}-URA	pCfB255 derivative, P_{PGK1}-*MdOSC1*^{C257Q}-T_{ADH1}, P_{TEF1}, T_{CYC1}, URA3	This study
p056-LOXP-MdOSC1^{C259A}-URA	Amp^{R}, pBR322 origin, P_{PGK1}-*MdOSC1*^{M255A}-T_{ADH1}, P_{TEF1}, T_{CYC1}, URA3	This study
p056-LOXP-MdOSC1^{C259G}-URA	pCfB255 derivative, P_{PGK1}-*MdOSC1*^{C259G}-T_{ADH1}, P_{TEF1}, T_{CYC1}, URA3	This study
p056-LOXP-MdOSC1^{C259I}-URA	pCfB255 derivative, P_{PGK1}-*MdOSC1*^{C259I}-T_{ADH1}, P_{TEF1}, T_{CYC1}, URA3	This study
p056-LOXP-MdOSC1^{C259L}-URA	pCfB255 derivative, P_{PGK1}-*MdOSC1*^{C259L}-T_{ADH1}, P_{TEF1}, T_{CYC1}, URA3	This study
p056-LOXP-MdOSC1^{C259P}-URA	pCfB255 derivative, P_{PGK1}-*MdOSC1*^{C259P}-T_{ADH1}, P_{TEF1}, T_{CYC1}, URA3	This study
p056-LOXP-MdOSC1^{C259V}-URA	pCfB255 derivative, P_{PGK1}-*MdOSC1*^{C259V}-T_{ADH1}, P_{TEF1}, T_{CYC1}, URA3	This study
p056-LOXP-MdOSC1^{C259F}-URA	pCfB255 derivative, P_{PGK1}-*MdOSC1*^{C259F}-T_{ADH1}, P_{TEF1}, T_{CYC1}, URA3	This study
p056-LOXP-MdOSC1^{C259W}-URA	pCfB255 derivative, P_{PGK1}-*MdOSC1*^{C259W}-T_{ADH1}, P_{TEF1}, T_{CYC1}, URA3	This study
p056-LOXP-MdOSC1^{C259Y}-URA	pCfB255 derivative, P_{PGK1}-*MdOSC1*^{C259Y}-T_{ADH1}, P_{TEF1}, T_{CYC1}, URA3	This study
p056-LOXP-MdOSC1^{C259D}-URA	pCfB255 derivative, P_{PGK1}-*MdOSC1*^{C259D}-T_{ADH1}, P_{TEF1}, T_{CYC1}, URA3	This study
p056-LOXP-MdOSC1^{C259E}-URA	pCfB255 derivative, P_{PGK1}-*MdOSC1*^{C259E}-T_{ADH1}, P_{TEF1}, T_{CYC1}, URA3	This study
p056-LOXP-MdOSC1^{C259R}-URA	pCfB255 derivative, P_{PGK1}-*MdOSC1*^{C259R}-T_{ADH1}, P_{TEF1}, T_{CYC1}, URA3	This study
p056-LOXP-MdOSC1^{C259H}-URA	pCfB255 derivative, P_{PGK1}-*MdOSC1*^{C259H}-T_{ADH1}, P_{TEF1}, T_{CYC1}, URA3	This study
p056-LOXP-MdOSC1^{C259K}-URA	pCfB255 derivative, P_{PGK1}-*MdOSC1*^{C259K}-T_{ADH1}, P_{TEF1}, T_{CYC1}, URA3	This study
p056-LOXP-MdOSC1^{C259S}-URA	pCfB255 derivative, P_{PGK1}-*MdOSC1*^{C259S}-T_{ADH1}, P_{TEF1}, T_{CYC1}, URA3	This study
p056-LOXP-MdOSC1^{C259T}-URA	pCfB255 derivative, P_{PGK1}-*MdOSC1*^{C259T}-T_{ADH1}, P_{TEF1}, T_{CYC1}, URA3	This study
p056-LOXP-MdOSC1^{C259M}-URA	pCfB255 derivative, P_{PGK1}-*MdOSC1*^{C259M}-T_{ADH1}, P_{TEF1}, T_{CYC1}, URA3	This study
p056-LOXP-MdOSC1^{C259N}-URA	pCfB255 derivative, P_{PGK1}-*MdOSC1*^{C259N}-T_{ADH1}, P_{TEF1}, T_{CYC1}, URA3	This study

p056-LOXP-MdOSC1^{C259Q}-URA	pCfB255 derivative, P_{PGK1}-*MdOSC1*^{C259Q}-T_{ADH1}, P_{TEF1}, T_{CYC1}, URA3	This study

Table S2 Strains used in this study

E. coli strains		
XL10-Gold	*TetrD(mcrA)183 D(mcrCB-hsdSMR-mrr)173 endA1 supE44 thi-1recA1 gyrA96 relA1 lac Hte [F' proAB lacIqZΔM15 Tn10 (Tet^r) Amy Cam^r]*	This study
SP1	*Saccharomyces cerevisiae* BY4742-TRP, dDNA:: P_{PGK1}-*tHMG1*-T_{ADH1} -P_{TEF1}-*LYS2*-T_{CYC1}	(Dai et al., 2014)
CTT1	SP1 derivative; P_{TEF1}-*GgbAS1*-T_{ADH1}, URA3	This study
CTT2	SP1 derivative; P_{TEF1}-*GgbAS1*^{M256A}-T_{ADH1}, URA3	This study
CTT3	SP1 derivative; P_{TEF1}-*GgbAS1*^{M256G}-T_{ADH1}, URA3	This study
CTT4	SP1 derivative; P_{TEF1}-*GgbAS1*^{M256I}-T_{ADH1}, URA3	This study
CTT5	SP1 derivative; P_{TEF1}-*GgbAS1*^{M256L}-T_{ADH1}, URA3	This study
CTT6	SP1 derivative; P_{TEF1}-*GgbAS1*^{M256P}-T_{ADH1}, URA3	This study
CTT7	SP1 derivative; P_{TEF1}-*GgbAS1*^{M256V}-T_{ADH1}, URA3	This study
CTT8	SP1 derivative; P_{TEF1}-*GgbAS1*^{M256F}-T_{ADH1}, URA3	This study
CTT9	SP1 derivative; P_{TEF1}-*GgbAS1*^{M256W}-T_{ADH1}, URA3	This study

CTT10	SP1 derivative; P_{TEF1}-*GgbAS1*^{M256Y}-T_{ADH1}, URA3	This study
CTT11	SP1 derivative; P_{TEF1}-*GgbAS1*^{M256D}-T_{ADH1}, URA3	This study
CTT12	SP1 derivative; P_{TEF1}-*GgbAS1*^{M256E}-T_{ADH1}, URA3	This study
CTT13	SP1 derivative; P_{TEF1}-*GgbAS1*^{M256R}-T_{ADH1}, URA3	This study
CTT14	SP1 derivative; P_{TEF1}-*GgbAS1*^{M256H}-T_{ADH1}, URA3	This study
CTT15	SP1 derivative; P_{TEF1}-*GgbAS1*^{M256K}-T_{ADH1}, URA3	This study
CTT16	SP1 derivative; P_{TEF1}-*GgbAS1*^{M256S}-T_{ADH1}, URA3	This study
CTT17	SP1 derivative; P_{TEF1}-*GgbAS1*^{M256T}-T_{ADH1}, URA3	This study
CTT18	SP1 derivative; P_{TEF1}-*GgbAS1*^{M256C}-T_{ADH1}, URA3	This study
CTT19	SP1 derivative; P_{TEF1}-*GgbAS1*^{M256N}-T_{ADH1}, URA3	This study
CTT20	SP1 derivative; P_{TEF1}-*GgbAS1*^{M256Q}-T_{ADH1}, URA3	This study
CTT21	SP1 derivative; P_{TEF1}-*GgbAS1*^{C258A}-T_{ADH1}, URA3	This study
CTT22	SP1 derivative; P_{TEF1}-*GgbAS1*^{C258G}-T_{ADH1}, URA3	This study

CTT23	SP1 derivative; *P_{TEF1}-GgbAS1^{C258I}-T_{ADH1}*, URA3	This study
CTT24	SP1 derivative; *P_{TEF1}-GgbAS1^{C258L}-T_{ADH1}*, URA3	This study
CTT25	SP1 derivative; *P_{TEF1}-GgbAS1^{C258P}-T_{ADH1}*, URA3	This study
CTT26	SP1 derivative; *P_{TEF1}-GgbAS1^{C258V}-T_{ADH1}*, URA3	This study
CTT27	SP1 derivative; *P_{TEF1}-GgbAS1^{C258F}-T_{ADH1}*, URA3	This study
CTT28	SP1 derivative; *P_{TEF1}-GgbAS1^{C258W}-T_{ADH1}*, URA3	This study
CTT29	SP1 derivative; *P_{TEF1}-GgbAS1^{C258Y}-T_{ADH1}*, URA3	This study
CTT30	SP1 derivative; *P_{TEF1}-GgbAS1^{C258D}-T_{ADH1}*, URA3	This study
CTT31	SP1 derivative; *P_{TEF1}-GgbAS1^{C258E}-T_{ADH1}*, URA3	This study
CTT32	SP1 derivative; *P_{TEF1}-GgbAS1^{C258R}-T_{ADH1}*, URA3	This study
CTT33	SP1 derivative; *P_{TEF1}-GgbAS1^{C258H}-T_{ADH1}*, URA3	This study
CTT34	SP1 derivative; *P_{TEF1}-GgbAS1^{C258K}-T_{ADH1}*, URA3	This study
CTT35	SP1 derivative; *P_{TEF1}-GgbAS1^{C258S}*-T_{ADH1}, URA3	This study

CTT36	SP1 derivative; P_{TEF1}-*GgbAS1*^{C258T}-T_{ADH1}, URA3	This study
CTT37	SP1 derivative; P_{TEF1}-*GgbAS1*^{C258M}-T_{ADH1}, URA3	This study
CTT38	SP1 derivative; P_{TEF1}-*GgbAS1*^{C258N}-T_{ADH1}, URA3	This study
CTT39	SP1 derivative; P_{TEF1}-*GgbAS1*^{C258Q}-T_{ADH1}, URA3	This study
CTT40	SP1 derivative; P_{TEF1}-*GgbAS1*^{C260A}-T_{ADH1}, URA3	This study
CTT41	SP1 derivative; P_{TEF1}-*GgbAS1*^{C260G}-T_{ADH1}, URA3	This study
CTT42	SP1 derivative; P_{TEF1}-*GgbAS1*^{C260I}-T_{ADH1}, URA3	This study
CTT43	SP1 derivative; P_{TEF1}-*GgbAS1*^{C260L}-T_{ADH1}, URA3	This study
CTT44	SP1 derivative; P_{TEF1}-*GgbAS1*^{C260P}-T_{ADH1}, URA3	This study
CTT45	SP1 derivative; P_{TEF1}-*GgbAS1*^{C260V}-T_{ADH1}, URA3	This study
CTT46	SP1 derivative; P_{TEF1}-*GgbAS1*^{C260F}-T_{ADH1}, URA3	This study
CTT47	SP1 derivative; P_{TEF1}-*GgbAS1*^{C260W}-T_{ADH1}, URA3	This study
CTT48	SP1 derivative; P_{TEF1}-*GgbAS1*^{C260Y}-T_{ADH1}, URA3	This study

CTT49	SP1 derivative; P_{TEF1}-$GgbAS1^{C260D}$-T_{ADH1}, URA3	This study
CTT50	SP1 derivative; P_{TEF1}-$GgbAS1^{C260E}$-T_{ADH1}, URA3	This study
CTT51	SP1 derivative; P_{TEF1}-$GgbAS1^{C260R}$-T_{ADH1}, URA3	This study
CTT52	SP1 derivative; P_{TEF1}-$GgbAS1^{C260H}$-T_{ADH1}, URA3	This study
CTT53	SP1 derivative; P_{TEF1}-$GgbAS1^{C260K}$-T_{ADH1}, URA3	This study
CTT54	SP1 derivative; P_{TEF1}-$GgbAS1^{C260S}$-T_{ADH1}, URA3	This study
CTT55	SP1 derivative; P_{TEF1}-$GgbAS1^{C260T}$-T_{ADH1}, URA3	This study
CTT56	SP1 derivative; P_{TEF1}-$GgbAS1^{C260M}$-T_{ADH1}, URA3	This study
CTT57	SP1 derivative; P_{TEF1}-$GgbAS1^{C260N}$-T_{ADH1}, URA3	This study
CTT58	SP1 derivative; P_{TEF1}-$GgbAS1^{C260Q}$-T_{ADH1}, URA3	This study
CTT59	SP1 derivative; P_{TEF1}-OEW-T_{ADH1}, URA3	This study
CTT60	SP1 derivative; P_{TEF1}-OEW^{M256A}-$\mathrm{T_{ADH1}}$, URA3	This study
CTT61	SP1 derivative; P_{TEF1}-OEW^{M256G}-T_{ADH1}, URA3	This study

CTT62	SP1 derivative; P_{TEF1}-OEW^{M256I}-T_{ADH1}, URA3	This study
CTT63	SP1 derivative; P_{TEF1}-OEW^{M256L}-T_{ADH1}, URA3	This study
CTT64	SP1 derivative; P_{TEF1}-OEW^{M256P}-T_{ADH1}, URA3	This study
CTT65	SP1 derivative; P_{TEF1}-OEW^{M256V}-T_{ADH1}, URA3	This study
CTT66	SP1 derivative; P_{TEF1}-OEW^{M256F}-T_{ADH1}, URA3	This study
CTT67	SP1 derivative; P_{TEF1}-OEW^{M256W}-T_{ADH1}, URA3	This study
CTT68	SP1 derivative; P_{TEF1}-OEW^{M256Y}-T_{ADH1}, URA3	This study
CTT69	SP1 derivative; P_{TEF1}-OEW^{M256D}-T_{ADH1}, URA3	This study
CTT70	SP1 derivative; P_{TEF1}-OEW^{M256E}-T_{ADH1}, URA3	This study
CTT71	SP1 derivative; P_{TEF1}-OEW^{M256R}-T_{ADH1}, URA3	This study
CTT72	SP1 derivative; P_{TEF1}-OEW^{M256H}-T_{ADH1}, URA3	This study
CTT73	SP1 derivative; P_{TEF1}-OEW^{M256K}-T_{ADH1}, URA3	This study
CTT74	SP1 derivative; P_{TEF1}-OEW^{M256S}-T_{ADH1}, URA3	This study

CTT75	SP1 derivative; P_{TEF1}-OEW^{M256T}-T_{ADH1}, URA3	This study
CTT76	SP1 derivative; P_{TEF1}-OEW^{M256M}-T_{ADH1}, URA3	This study
CTT77	SP1 derivative; P_{TEF1}-OEW^{M256N}-T_{ADH1}, URA3	This study
CTT78	SP1 derivative; P_{TEF1}-OEW^{M256Q}-T_{ADH1}, URA3	This study
CTT79	SP1 derivative; P_{TEF1}-OEW^{C258A}-T_{ADH1}, URA3	This study
CTT80	SP1 derivative; P_{TEF1}-OEW^{C258G}-T_{ADH1}, URA3	This study
CTT81	SP1 derivative; P_{TEF1}-OEW^{C258I}-T_{ADH1}, URA3	This study
CTT82	SP1 derivative; P_{TEF1}-OEW^{C258L}-T_{ADH1}, URA3	This study
CTT83	SP1 derivative; P_{TEF1}-OEW^{C258P}-T_{ADH1}, URA3	This study
CTT84	SP1 derivative; P_{TEF1}-OEW^{C258V}-T_{ADH1}, URA3	This study
CTT85	SP1 derivative; P_{TEF1}-OEW^{C258F}-T_{ADH1}, URA3	This study
CTT86	SP1 derivative; P_{TEF1}-OEW^{C258W}-T_{ADH1}, URA3	This study
CTT87	SP1 derivative; P_{TEF1}-OEW^{C258Y}-T_{ADH1}, URA3	This study

CTT88	SP1 derivative; P_{TEF1}-OEW^{C258D}-T_{ADH1}, URA3	This study
CTT89	SP1 derivative; P_{TEF1}-OEW^{C258E}-T_{ADH1}, URA3	This study
CTT90	SP1 derivative; P_{TEF1}-OEW^{C258R}-T_{ADH1}, URA3	This study
CTT91	SP1 derivative; P_{TEF1}-OEW^{C258H}-T_{ADH1}, URA3	This study
CTT92	SP1 derivative; P_{TEF1}-OEW^{C258K}-T_{ADH1}, URA3	This study
CTT93	SP1 derivative; P_{TEF1}-OEW^{C258S}-T_{ADH1}, URA3	This study
CTT94	SP1 derivative; P_{TEF1}-OEW^{C258T}-T_{ADH1}, URA3	This study
CTT95	SP1 derivative; P_{TEF1}-OEW^{C258M}-T_{ADH1}, URA3	This study
CTT96	SP1 derivative; P_{TEF1}-OEW^{C258N}-T_{ADH1}, URA3	This study
CTT97	SP1 derivative; P_{TEF1}-OEW^{C258Q}-T_{ADH1}, URA3	This study
CTT98	SP1 derivative; P_{TEF1}-OEW^{C260A}-T_{ADH1}, URA3	This study
CTT99	SP1 derivative; P_{TEF1}-OEW^{C260G}-T_{ADH1}, URA3	This study
CTT100	SP1 derivative; P_{TEF1}-OEW^{C260I}-T_{ADH1}, URA3	This study

CTT101	SP1 derivative; P_{TEF1}-OEW^{C260L}-T_{ADH1}, URA3	This study
CTT102	SP1 derivative; P_{TEF1}-OEW^{C260P}-T_{ADH1}, URA3	This study
CTT103	SP1 derivative; P_{TEF1}-OEW^{C260V}-T_{ADH1}, URA3	This study
CTT104	SP1 derivative; P_{TEF1}-OEW^{C260F}-T_{ADH1}, URA3	This study
CTT105	SP1 derivative; P_{TEF1}-OEW^{C260W}-T_{ADH1}, URA3	This study
CTT106	SP1 derivative; P_{TEF1}-OEW^{C260Y}-T_{ADH1}, URA3	This study
CTT107	SP1 derivative; P_{TEF1}-OEW^{C260D}-T_{ADH1}, URA3	This study
CTT108	SP1 derivative; P_{TEF1}-OEW^{C260E}-T_{ADH1}, URA3	This study
CTT109	SP1 derivative; P_{TEF1}-OEW^{C260R}-T_{ADH1}, URA3	This study
CTT110	SP1 derivative; P_{TEF1}-OEW^{C260H}-T_{ADH1}, URA3	This study
CTT111	SP1 derivative; P_{TEF1}-OEW^{C260K}-T_{ADH1}, URA3	This study
CTT112	SP1 derivative; P_{TEF1}-OEW^{C260S}-T_{ADH1}, URA3	This study
CTT113	SP1 derivative; P_{TEF1}-OEW^{C260T}-T_{ADH1}, URA3	This study

CTT114	SP1 derivative; P_{TEF1}-OEW^{C260M}-T_{ADH1}, URA3	This study
CTT115	SP1 derivative; P_{TEF1}-OEW^{C260N}-T_{ADH1}, URA3	This study
CTT116	SP1 derivative; P_{TEF1}-OEW^{C260Q}-T_{ADH1}, URA3	This study
CTT117	SP1 derivative; P_{TEF1}-*MdOSC1*-T_{ADH1}, URA3	This study
CTT118	SP1 derivative; P_{TEF1}-$MdOSC1^{M255A}$-T_{ADH1}, URA3	This study
CTT119	SP1 derivative; P_{TEF1}-$MdOSC1^{M255G}$-T_{ADH1}, URA3	This study
CTT120	SP1 derivative; P_{TEF1}-$MdOSC1^{M255I}$-T_{ADH1}, URA3	This study
CTT121	SP1 derivative; P_{TEF1}-$MdOSC1^{M255L}$-T_{ADH1}, URA3	This study
CTT122	SP1 derivative; P_{TEF1}-$MdOSC1^{M255P}$-T_{ADH1}, URA3	This study
CTT123	SP1 derivative; P_{TEF1}-$MdOSC1^{M255V}$-T_{ADH1}, URA3	This study
CTT124	SP1 derivative; P_{TEF1}-$MdOSC1^{M255F}$-T_{ADH1}, URA3	This study
CTT125	SP1 derivative; P_{TEF1}-$MdOSC1^{M255W}$-T_{ADH1}, URA3	This study
CTT126	SP1 derivative; P_{TEF1}-$MdOSC1^{M255Y}$-T_{ADH1}, URA3	This study

CTT127	SP1 derivative; P_{TEF1}-*MdOSC1*^{M255D}-T_{ADH1}, URA3	This study
CTT128	SP1 derivative; P_{TEF1}-*MdOSC1*^{M255E}-T_{ADH1}, URA3	This study
CTT129	SP1 derivative; P_{TEF1}-*MdOSC1*^{M255R}-T_{ADH1}, URA3	This study
CTT130	SP1 derivative; P_{TEF1}-*MdOSC1*^{M255H}-T_{ADH1}, URA3	This study
CTT131	SP1 derivative; P_{TEF1}-*MdOSC1*^{M255K}-T_{ADH1}, URA3	This study
CTT132	SP1 derivative; P_{TEF1}-*MdOSC1*^{M255S}-T_{ADH1}, URA3	This study
CTT133	SP1 derivative; P_{TEF1}-*MdOSC1*^{M255T}-T_{ADH1}, URA3	This study
CTT134	SP1 derivative; P_{TEF1}-*MdOSC1*^{M255M}-T_{ADH1}, URA3	This study
CTT135	SP1 derivative; P_{TEF1}-*MdOSC1*^{M255N}-T_{ADH1}, URA3	This study
CTT136	SP1 derivative; P_{TEF1}-*MdOSC1*^{M255Q}-T_{ADH1}, URA3	This study
CTT137	SP1 derivative; P_{TEF1}-*MdOSC1*^{C257A}-T_{ADH1}, URA3	This study
CTT138	SP1 derivative; P_{TEF1}-*MdOSC1*^{C257G}-T_{ADH1}, URA3	This study
CTT139	SP1 derivative; P_{TEF1}-MdOSC1^{C257I}-T_{ADH1}, URA3	This study

CTT140	SP1 derivative; P_{TEF1}-MdOSC1^{C257L}-T_{ADH1}, URA3	This study
CTT141	SP1 derivative; P_{TEF1}-MdOSC1^{C257P}-T_{ADH1}, URA3	This study
CTT142	SP1 derivative; P_{TEF1}-MdOSC1^{C257V}-T_{ADH1}, URA3	This study
CTT143	SP1 derivative; P_{TEF1}-MdOSC1^{C257F}-T_{ADH1}, URA3	This study
CTT144	SP1 derivative; P_{TEF1}-MdOSC1^{C257W}-T_{ADH1}, URA3	This study
CTT145	SP1 derivative; P_{TEF1}-MdOSC1^{C257Y}-T_{ADH1}, URA3	This study
CTT146	SP1 derivative; P_{TEF1}-MdOSC1^{C257D}-T_{ADH1}, URA3	This study
CTT147	SP1 derivative; P_{TEF1}-$MdOSC1^{C257E}$-T_{ADH1}, URA3	This study
CTT148	SP1 derivative; P_{TEF1}-$MdOSC1^{C257R}$-T_{ADH1}, URA3	This study
CTT149	SP1 derivative; P_{TEF1}-$MdOSC1^{C257H}$-T_{ADH1}, URA3	This study
CTT150	SP1 derivative; P_{TEF1}-$MdOSC1^{C257K}$-T_{ADH1}, URA3	This study
CTT151	SP1 derivative; P_{TEF1}-$MdOSC1^{C257S}$-T_{ADH1}, URA3	This study
CTT152	SP1 derivative; P_{TEF1}-$MdOSC1^{C257T}$-T_{ADH1}, URA3	This study

CTT153	SP1 derivative; P_{TEF1}-$MdOSC1^{C257M}$-T_{ADH1}, URA3	This study
CTT154	SP1 derivative; P_{TEF1}-$MdOSC1^{C257N}$-T_{ADH1}, URA3	This study
CTT155	SP1 derivative; P_{TEF1}-$MdOSC1^{C257Q}$-T_{ADH1}, URA3	This study
CTT156	SP1 derivative; P_{TEF1}-$MdOSC1^{C2597A}$-T_{ADH1}, URA3	This study
CTT157	SP1 derivative; P_{TEF1}-$MdOSC1^{C2597G}$-T_{ADH1}, URA3	This study
CTT158	SP1 derivative; P_{TEF1}-$MdOSC1^{C2597I}$-T_{ADH1}, URA3	This study
CTT159	SP1 derivative; P_{TEF1}-$MdOSC1^{C2597L}$-T_{ADH1}, URA3	This study
CTT160	SP1 derivative; P_{TEF1}-$MdOSC1^{C2597P}$-T_{ADH1}, URA3	This study
CTT161	SP1 derivative; P_{TEF1}-$MdOSC1^{C2597V}$-T_{ADH1}, URA3	This study
CTT162	SP1 derivative; P_{TEF1}-$MdOSC1^{C2597F}$-T_{ADH1}, URA3	This study
CTT163	SP1 derivative; P_{TEF1}-$MdOSC1^{C2597W}$-T_{ADH1}, URA	This study
CTT164	SP1 derivative; P_{TEF1}-$MdOSC1^{C2597Y}$-T_{ADH1}, URA3	This study
CTT165	SP1 derivative; P_{TEF1}-$MdOSC1^{C2597D}$-T_{ADH1}, URA3	This study

CTT166	SP1 derivative; P_{TEF1}-*MdOSC1*^{C2597E}-T_{ADH1}, URA3	This study
CTT167	SP1 derivative; P_{TEF1}-*MdOSC1*^{C2597R}-T_{ADH1}, URA3	This study
CTT168	SP1 derivative; P_{TEF1}-*MdOSC1*^{C2597H}-T_{ADH1}, URA3	This study
CTT169	SP1 derivative; P_{TEF1}-*MdOSC1*^{C2597K}-T_{ADH1}, URA3	This study
CTT170	SP1 derivative; P_{TEF1}-*MdOSC1*^{C2597S}-T_{ADH1}, URA3	This study
CTT171	SP1 derivative; P_{TEF1}-*MdOSC1*^{C2597T}-T_{ADH1}, URA3	This study
CTT172	SP1 derivative; P_{TEF1}-*MdOSC1*^{C2597M}-T_{ADH1}, URA3	This study
CTT173	SP1 derivative; P_{TEF1}-*MdOSC1*^{C2597N}-T_{ADH1}, URA3	This study
CTT174	SP1 derivative; P_{TEF1}-*MdOSC1*^{C2597Q}-T_{ADH1}, URA3	This study
CTT175	SP1 derivative; P_{TEF1}-GgbAS1$^{M256Q/C258A}$-T_{ADH1}, URA3	This study
CTT176	SP1 derivative; P_{TEF1}-GgbAS1$^{M256Q/C258V}$-T_{ADH1}, URA3	This study
CTT177	SP1 derivative; P_{TEF1}-*GgbAS1*$^{M256A/C258A}$-T_{ADH1}, URA3	This study
CTT178	SP1 derivative; P_{TEF1}-GgbAS1$^{M256A/C258V}$-T_{ADH1}, URA3	This study

CTT179	SP1 derivative; P_{TEF1}-*GgbAS1*$^{M256Q/C260W}$-T_{ADH1}, URA3	This study
CTT180	SP1 derivative; P_{TEF1}-*GgbAS1*$^{M256Q/C260H}$-T_{ADH1}, URA3	This study
CTT181	SP1 derivative; P_{TEF1}-*GgbAS1*$^{M256A/C260W}$-T_{ADH1}, URA3	This study
CTT182	SP1 derivative; P_{TEF1}-*GgbAS1*$^{M256A/C260H}$-T_{ADH1}, URA3	This study
CTT183	SP1 derivative; P_{TEF1}-*GgbAS1*$^{C258A/C260W}$-T_{ADH1}, URA3	This study
CTT184	SP1 derivative; P_{TEF1}-*GgbAS1*$^{C258A/C260H}$-T_{ADH1}, URA3	This study
CTT185	SP1 derivative; P_{TEF1}-*GgbAS1*$^{C258V/C260W}$-T_{ADH1}, URA3	This study
CTT186	SP1 derivative; P_{TEF1}-*GgbAS1*$^{C258V/C260H}$-T_{ADH1}, URA3	This study
CTT187	SP1 derivative; P_{TEF1}-*GgbAS1*$^{M256A/C258A/C260W}$-T_{ADH1}, URA3	This study
CTT188	SP1 derivative; P_{TEF1}-*GgbAS1*$^{M256A/C258V/C260W}$-T_{ADH1}, URA3	This study
CTT189	SP1 derivative; P_{TEF1}-*GgbAS1*$^{M256A/C258A/C260H}$-T_{ADH1}, URA3	This study
CTT190	SP1 derivative; P_{TEF1}-GgbAS1$^{M256A/C258V/C260H}$-T_{ADH1}, URA3	This study
CTT191	SP1 derivative; P_{TEF1}-*GgbAS1*$^{M256Q/C258A/C260W}$-T_{ADH1}, URA3	This study

CTT192	SP1 derivative; P_{TEF1}-*GgbAS1*$^{M256Q/C258V/C260W}$-T_{ADH1}, URA3	This study
CTT193	SP1 derivative; P_{TEF1}-*GgbAS1*$^{M256Q/C258A/C260H}$-T_{ADH1}, URA3	This study
CTT194	SP1 derivative; P_{TEF1}-*GgbAS1*$^{M256Q/C258V/C260H}$-T_{ADH1}, URA3	This study
CTT195	SP1 derivative; P_{TEF1}-*OEW*$^{M255V/C259A}$-T_{ADH1}, URA3	This study
CTT196	SP1 derivative; P_{TEF1}-*OEW*$^{M255L/C259A}$-T_{ADH1}, URA3	This study
CTT197	SP1 derivative; P_{TEF1}-*OEW*$^{M255C/C259A}$-T_{ADH1}, URA3	This study
CTT198	SP1 derivative; P_{TEF1}-*OEW*$^{M255I/C259A}$-T_{ADH1}, URA3	This study
CTT199	SP1 derivative; P_{TEF1}-*OEW*$^{M255Y/C259A}$-T_{ADH1}, URA3	This study
CTT200	SP1 derivative; P_{TEF1}-*OEW*$^{M255V/C259G}$-T_{ADH1}, URA3	This study
CTT201	SP1 derivative; P_{TEF1}-*OEW*$^{M255L/C259G}$-T_{ADH1}, URA3	This study
CTT202	SP1 derivative; P_{TEF1}-*OEW*$^{M255V/C259G}$-T_{ADH1}, URA3	This study
CTT203	SP1 derivative; P_{TEF1}-*OEW*$^{M255I/C259G}$-T_{ADH1}, URA3	This study
CTT204	SP1 derivative; P_{TEF1}-*OEW*$^{M256Y/C259G}$-T_{ADH1}, URA3	This study

CTT205	SP1 derivative; P_{TEF1}-*MdOSC1* N11T/P250H/P373A/M255T-T_{ADH1}, URA3	This study

Table S3 Primers used in this study

Name	Sequence	Application	Length
GgbAS1F	ATGTGGAGATTAAAGATTGCTGAAGG		
GgbAS1R	TCAAGTCAAGCAAACTGGTGTAGATG		
GgbAS1BBF	CCATCTACACCAGTTTGCTTGACTTGAATC GCTCGGCATGCCGGTA		
GgbAS1BBR	ACCTTCAGCAATCTTTAATCTCCACATACCT GGTCTTAGATTAGATTGCTATGC		
OEWF	TTAAGTTTGTGCATGCAATATTCTTCT		
OEWR	CAACTTGATACCTCTATGGATAGGACC		
OEWBBF	GTCCTATCCATAGAGGTATCAAGTTGACCT GGTCTTAGATTAGATTGCTATGC		
OEWBBR	GAAGAATATTGCATGCACAAACTTAAATCG CTCGGCATGCCGGTAGA		
MdOSC1F	ATGTGGAAGATTAAGTTTGGTGAAGGA		
MdOSC1R	TCAAGCAATTTTCTTAATTGGCAATG		
MdOSC1BBF	CATTGCCAATTAAGAAAATTGCTTGATCGG CATGCCGGTAGAGGTGTGGT		
MdOSC1BBR	CTTCACCAAACTTAATCTTCCACATGGTATA TCTCCTTCTTAAAGCTTAGATTAGATTGC		

GgbAS1 M256AF	ATCCTGCAAAAGCTTGGTGTTATTGCAGAT TGGTTTA
GgbAS1 M256AR	TGCAATAACACCAAGCTTTTGCAGGATGCA TTG
GgbAS1 M256GF	ATCCTGCAAAAGGTTGGTGTTATTGCAGAT TGGTTTA
GgbAS1 M256GR	TGCAATAACACCAACCTTTTGCAGGATGCA TTG
GgbAS1 M256IF	ATCCTGCAAAAATTTGGTGTTATTGCAGATT GGTTTA
GgbAS1 M256IR	TGCAATAACACCAAATTTTTGCAGGATGCA TTG
GgbAS1 M256LF	ATCCTGCAAAACTTTGGTGTTATTGCAGAT TGGTTTA
GgbAS1 M256LR	TGCAATAACACCAAAGTTTTGCAGGATGCA TTG
GgbAS1 M256PF	ATCCTGCAAAACCTTGGTGTTATTGCAGAT TGGTTTA
GgbAS1 M256PR	TGCAATAACACCAAGGTTTTGCAGGATGCA TTG
GgbAS1 M256VF	ATCCTGCAAAAGTTTGGTGTTATTGCAGAT TGGTTTA
GgbAS1 M256VR	TGCAATAACACCAAACTTTTGCAGGATGCA TTG
GgbAS1 M256FF	ATCCTGCAAAATTTTGGTGTTATTGCAGATT GGTTTA
GgbAS1 M256FR	TGCAATAACACCAAAATTTTGCAGGATGCA TTG

GgbAS1 M256WF	ATCCTGCAAAATGGTGGTGTTATTGCAGATTGGTTTA
GgbAS1 M256WR	TGCAATAACACCACCATTTTGCAGGATGCATTG
GgbAS1 M256YF	ATCCTGCAAAATATTGGTGTTATTGCAGATTGGTTTA
GgbAS1 M256YR	TGCAATAACACCAATATTTTGCAGGATGCATTG
GgbAS1 M256DF	ATCCTGCAAAAGATTGGTGTTATTGCAGATTGGTTTA
GgbAS1 M256DR	TGCAATAACACCAATCTTTTGCAGGATGCATTG
GgbAS1 M256EF	ATCCTGCAAAAGAATGGTGTTATTGCAGATTGGTTTA
GgbAS1 M256ER	TGCAATAACACCATTCTTTTGCAGGATGCATTG
GgbAS1 M256RF	ATCCTGCAAAACGTTGGTGTTATTGCAGATTGGTTTA
GgbAS1 M256RR	TGCAATAACACCAACGTTTTGCAGGATGCATTG
GgbAS1 M256HF	ATCCTGCAAAACATTGGTGTTATTGCAGATTGGTTTA
GgbAS1 M256HR	TGCAATAACACCAATGTTTTGCAGGATGCATTG
GgbAS1 M256KF	ATCCTGCAAAAAAATGGTGTTATTGCAGATTGGTTTA
GgbAS1 M256KR	TGCAATAACACCATTTTTTTGCAGGATGCATTG

GgbAS1 M256SF	ATCCTGCAAAATCTTGGTGTTATTGCAGATT GGTTTA
GgbAS1 M256SR	TGCAATAACACCAAGATTTTGCAGGATGCA TTG
GgbAS1 M256TF	ATCCTGCAAAAACTTGGTGTTATTGCAGAT TGGTTTA
GgbAS1 M256TR	TGCAATAACACCAAGTTTTTGCAGGATGCA TTG
GgbAS1 M256CF	ATCCTGCAAAATGTTGGTGTTATTGCAGATT GGTTTA
GgbAS1 M256CR	TGCAATAACACCAACATTTTGCAGGATGCA TTG
GgbAS1 M256NF	ATCCTGCAAAAAATTGGTGTTATTGCAGAT TGGTTTA
GgbAS1 M256NR	TGCAATAACACCAATTTTTTGCAGGATGCA TTG
GgbAS1 M256QF	ATCCTGCAAAACAATGGTGTTATTGCAGAT TGGTTTA
GgbAS1 M256QR	TGCAATAACACCATTGTTTTGCAGGATGCA TTG
GgbAS1 C258AF	ATGTGGGCTTATTGCAGATTGGTTTACATGC
GgbAS1 C258AR	TCTGCAATAAGCCCACATTTTTGCAGGATG C
GgbAS1 C258GF	ATGTGGGGTTATTGCAGATTGGTTTACATGC
GgbAS1 C258GR	TCTGCAATAACCCCACATTTTTGCAGGATG C

GgbAS1 C258IF	ATGTGGATCTATTGCAGATTGGTTTACATGC
GgbAS1 C258IR	TCTGCAATAGATCCACATTTTTGCAGGATG<br>C
GgbAS1 C258LF	ATGTGGTTATATTGCAGATTGGTTTACATGC
GgbAS1 C258LR	TCTGCAATATAACCACATTTTTGCAGGATGC
GgbAS1 C258PF	ATGTGGCCTTATTGCAGATTGGTTTACATGC
GgbAS1 C258PR	TCTGCAATAAGGCCACATTTTTGCAGGATG<br>C
GgbAS1 C258VF	ATGTGGGTTTATTGCAGATTGGTTTACATGC
GgbAS1 C258VR	TCTGCAATAAACCCACATTTTTGCAGGATG<br>C
GgbAS1 C258FF	ATGTGGTTTTATTGCAGATTGGTTTACATGC
GgbAS1 C258FR	TCTGCAATAAAACCACATTTTTGCAGGATG<br>C
GgbAS1 C258WF	ATGTGGTGGTATTGCAGATTGGTTTACATGC
GgbAS1 C258WR	TCTGCAATACCACCACATTTTTGCAGGATG<br>C
GgbAS1 C258YF	ATGTGGTATTATTGCAGATTGGTTTACATGC
GgbAS1 C258YR	TCTGCAATAATACCACATTTTTGCAGGATGC

GgbAS1 C258DF	ATGTGGGATTATTGCAGATTGGTTTACATGC
GgbAS1 C258DR	TCTGCAATAATCCCACATTTTTGCAGGATGC
GgbAS1 C258EF	ATGTGGGAATATTGCAGATTGGTTTACATGC
GgbAS1 C258ER	TCTGCAATATTCCCACATTTTTGCAGGATGC
GgbAS1 C258RF	ATGTGGCGTTATTGCAGATTGGTTTACATGC
GgbAS1 C258RR	TCTGCAATAACGCCACATTTTTGCAGGATGC
GgbAS1 C258HF	ATGTGGCATTATTGCAGATTGGTTTACATGC
GgbAS1 C258HR	TCTGCAATAATGCCACATTTTTGCAGGATGC
GgbAS1 C258KF	ATGTGGAAATATTGCAGATTGGTTTACATGC
GgbAS1 C258KR	TCTGCAATATTTCCACATTTTTGCAGGATGC
GgbAS1 C258SF	ATGTGGAGTTATTGCAGATTGGTTTACATGC
GgbAS1 C258SR	TCTGCAATAACTCCACATTTTTGCAGGATGC
GgbAS1 C258TF	ATGTGGACTTATTGCAGATTGGTTTACATGC
GgbAS1 C258TR	TCTGCAATAAGTCCACATTTTTGCAGGATGC

GgbAS1 C258MF	ATGTGGATGTATTGCAGATTGGTTTACATGC
GgbAS1 C258MR	TCTGCAATACATCCACATTTTTGCAGGATGC
GgbAS1 C258NF	ATGTGGAATTATTGCAGATTGGTTTACATGC
GgbAS1 C258NR	TCTGCAATAATTCCACATTTTTGCAGGATGC
GgbAS1 C258QF	ATGTGGCAATATTGCAGATTGGTTTACATGC
GgbAS1 C258QR	TCTGCAATATTGCCACATTTTTGCAGGATGC
GgbAS1 C260AF	TGTGGTGTTATGCTAGATTGGTTTACATGCCA
GgbAS1 C260AR	ATGTAAACCAATCTAGCATAACACCACATTTTTGC
GgbAS1 C260GF	TGTGGTGTTATGGTAGATTGGTTTACATGCCA
GgbAS1 C260GR	ATGTAAACCAATCTACCATAACACCACATTTTTGC
GgbAS1 C260IF	TGTGGTGTTATATTAGATTGGTTTACATGCCA
GgbAS1 C260IR	ATGTAAACCAATCTAATATAACACCACATTTTTGC
GgbAS1 C260LF	TGTGGTGTTATCTTAGATTGGTTTACATGCCA
GgbAS1 C260LR	ATGTAAACCAATCTAAGATAACACCACATTTTTGC

GgbAS1 C260PF	TGTGGTGTTATCCTAGATTGGTTTACATGCCA
GgbAS1 C260PR	ATGTAAACCAATCTAGGATAACACCACATTTTTGC
GgbAS1 C260VF	TGTGGTGTTATGTTAGATTGGTTTACATGCCA
GgbAS1 C260VR	ATGTAAACCAATCTAACATAACACCACATTTTTGC
GgbAS1 C260FF	TGTGGTGTTATTTTAGATTGGTTTACATGCCA
GgbAS1 C260FR	ATGTAAACCAATCTAAAATAACACCACATTTTTGC
GgbAS1 C260WF	TGTGGTGTTATTGGAGATTGGTTTACATGCCA
GgbAS1 C260WR	ATGTAAACCAATCTCCAATAACACCACATTTTTGC
GgbAS1 C260YF	TGTGGTGTTATTATAGATTGGTTTACATGCCA
GgbAS1 C260YR	ATGTAAACCAATCTATAATAACACCACATTTTTGC
GgbAS1 C260DF	TGTGGTGTTATGATAGATTGGTTTACATGCCA
GgbAS1 C260DR	ATGTAAACCAATCTATCATAACACCACATTTTTGC
GgbAS1 C260EF	TGTGGTGTTATGAAAGATTGGTTTACATGCCA
GgbAS1 C260ER	ATGTAAACCAATCTTTCATAACACCACATTTTTGC

GgbAS1 C260RF	TGTGGTGTTATCGTAGATTGGTTTACATGCCA
GgbAS1 C260RR	ATGTAAACCAATCTACGATAACACCACATTTTTGC
GgbAS1 C260HF	TGTGGTGTTATCATAGATTGGTTTACATGCCA
GgbAS1 C260HR	ATGTAAACCAATCTATGATAACACCACATTTTTGC
GgbAS1 C260KF	TGTGGTGTTATAAAAGATTGGTTTACATGCCA
GgbAS1 C260KR	ATGTAAACCAATCTTTTATAACACCACATTTTTGC
GgbAS1 C260SF	TGTGGTGTTATTCTAGATTGGTTTACATGCCA
GgbAS1 C260SR	ATGTAAACCAATCTAGAATAACACCACATTTTTGC
GgbAS1 C260TF	TGTGGTGTTATACTAGATTGGTTTACATGCCA
GgbAS1 C260TR	ATGTAAACCAATCTAGTATAACACCACATTTTTGC
GgbAS1 C260MF	TGTGGTGTTATATGAGATTGGTTTACATGCCA
GgbAS1 C260MR	ATGTAAACCAATCTCATATAACACCACATTTTTGC
GgbAS1 C260NF	TGTGGTGTTATAATAGATTGGTTTACATGCCA
GgbAS1 C260NR	ATGTAAACCAATCTATTATAACACCACATTTTTGC

GgbAS1 C260QF	TGTGGTGTTATCAAAGATTGGTTTACATGCCA
GgbAS1 C260QR	ATGTAAACCAATCTTTGATAACACCACATTTTTGC
GgbAS1M256QC258V-F	AAACAATGGGTTTATTGCAGATTGGTTTACATGCCAATGTCTT
GgbAS1M256QC258V-R	AATCTGCAATAAACCCATTGTTTTGCAGGATGCATTGGCAAG
GgbAS1M256QC258A-F	AAACAATGGGCTTATTGCAGATTGGTTTACATGCCAATGTCTT
GgbAS1M256QC258A-R	AATCTGCAATAAGCCCATTGTTTTGCAGGATGCATTGGCAAG
GgbAS1M256AC258A-F	AAAGCTTGGGCTTATTGCAGATTGGTTTACATGCCAATGTCTT
GgbAS1M256AC258A-R	AATCTGCAATAAGCCCAAGCTTTTGCAGGATGCATTGGCAAG
GgbAS1M256AC258V-F	AAAGCTTGGGTTTATTGCAGATTGGTTTACATGCCAATGTCTT
GgbAS1M256AC258V-R	AATCTGCAATAAACCCAAGCTTTTGCAGGATGCATTGGCAAG
GgbAS1M256QC260W-F	AAACAATGGTGTTATTGGAGATTGGTTTACATGCCAATGTCTT
GgbAS1M256QC260W-R	AATCTCCAATAACACCATTGTTTTGCAGGATGCATTGGCAAG
GgbAS1M256QC260H-F	AAACAATGGTGTTATCATAGATTGGTTTACATGCCAATGTCTT
GgbAS1M256QC260H-R	AATCTATGATAACACCATTGTTTTGCAGGATGCATTGGCAAG

GgbAS1M256AC260H-F	AAAGCTTGGTGTTATCATAGATTGGTTTACATGCCAATGTCTT
GgbAS1M256AC260H-R	AATCTATGATAACACCAAGCTTTTGCAGGATGCATTGGCAAG
GgbAS1M256AC260W-F	AAAGCTTGGTGTTATTGGAGATTGGTTTACATGCCAATGTCTT
GgbAS1M256AC260W-R	AATCTCCAATAACACCAAGCTTTTGCAGGATGCATTGGCAAG
GgbAS1C258AC260W-F	AAAATGTGGGCTTATTGGAGATTGGTTTACATGCCAATGTCTT
GgbAS1C258AC260W-R	AATCTCCAATAAGCCCACATTTTTGCAGGATGCATTGGCAAG
GgbAS1C258AC260H-F	AAAATGTGGGCTTATCATAGATTGGTTTACATGCCAATGTCTT
GgbAS1C258AC260H-R	AATCTATGATAAGCCCACATTTTTGCAGGATGCATTGGCAAG
GgbAS1C258VC260H-F	AAAATGTGGGTTTATCATAGATTGGTTTACATGCCAATGTCTT
GgbAS1C258VC260H-R	AATCTATGATAAACCCACATTTTTGCAGGATGCATTGGCAAG
GgbAS1C258VC260W-F	AAAATGTGGGTTTATTGGAGATTGGTTTACATGCCAATGTCTT
GgbAS1C258VC260W-R	AATCTCCAATAAACCCACATTTTTGCAGGATGCATTGGCAAG
ggbas M256AC258AC260W-F	GCATCCTGCAAAAGCTTGGGCTTATTGGAGATTGGTTTACATGCCAATGTCTT
GgbAS1 M256AC258AC260W-R	ACCAATCTCCAATAAGCCCAAGCTTTTGCAGGATGCATTGGCAAGAAACTAG

GgbAS1 M256AC258VC260 W-F	GCATCCTGCAAAAGCTTGGGTTTATTGGAGATTGGTTTACATGCCAATGTCTT
GgbAS1 M256AC258VC260 W-R	ACCAATCTCCAATAAACCCAAGCTTTTGCAGGATGCATTGGCAAGAAACTAG
GgbAS1 M256AC258IC260 W-F	GCATCCTGCAAAAGCTTGGATTTATTGGAGATTGGTTTACATGCCAATGTCTT
GgbAS1 M256AC258IC260 W-R	ACCAATCTCCAATAAATCCAAGCTTTTGCAGGATGCATTGGCAAGAAACTAG
GgbAS1 M256AC258VC260 L-F	GCATCCTGCAAAAGCTTGGGTTTATCTTAGATTGGTTTACATGCCAATGTCTT
GgbAS1 M256AC258VC260 L-R	ACCAATCTAAGATAAACCCAAGCTTTTGCAGGATGCATTGGCAAGAAACTAG
GgbAS1 M256AC258VC260 E-F	GCATCCTGCAAAAGCTTGGGTTTATGAAAGATTGGTTTACATGCCAATGTCTT
GgbAS1 M256AC258VC260 E-R	CTAGTTTCTTGCCAATGCATCCTGCAAAAGCTTGGGTTTATGAAAGATTGGT
GgbAS1 M256AC258VC260 T-F	GCATCCTGCAAAAGCTTGGGTTTATACTAGATTGGTTTACATGCCAATGTCTT
GgbAS1 M256AC258VC260 T-R	CTAGTTTCTTGCCAATGCATCCTGCAAAAGCTTGGGTTTATACTAGATTGGT
GgbAS1 M256HC258VC260 W-F	GCATCCTGCAAAACATTGGGTTTATTGGAGATTGGTTTACATGCCAATGTCTT
GgbAS1 M256HC258VC260 W-R	CTAGTTTCTTGCCAATGCATCCTGCAAAACATTGGGTTTATTGGAGATTGGT
GgbAS1 M256PC258VC260 W-F	GCATCCTGCAAAACCTTGGGTTTATTGGAGATTGGTTTACATGCCAATGTCTT

GgbAS1	CTAGTTTCTTGCCAATGCATCCTGCAAAAC
M256PC258VC260	CTTGGGTTTATTGGAGATTGGT
W-R	
GgbAS1	CCTGCAAAACAATGGGTTTATTGGAGATTG
M256QC258VC260	GTTTACATGCCAATGTCTT
W-F	
GgbAS1	AATCTCCAATAAACCCATTGTTTTGCAGGA
M256QC258VC260	TGCATTGGCAAGAAACTAG
W-R	
GgbAS1	CCTGCAAAACAATGGGCTTATTGGAGATTG
M256QC258AC260	GTTTACATGCCAATGTCTT
W-F	
GgbAS1	AATCTCCAATAAGCCCATTGTTTTGCAGGA
M256QC258AC260	TGCATTGGCAAGAAACTAG
W-R	
GgbAS1	CCTGCAAAACAATGGGTTTATCATAGATTG
M256QC258VC260	GTTTACATGCCAATGTCTT
H-F	
GgbAS1	AATCTATGATAAACCCATTGTTTTGCAGGAT
M256QC258VC260	GCATTGGCAAGAAACTAG
H-R	
GgbAS1	CCTGCAAAACAATGGGCTTATCATAGATTG
M256QC258AC260	GTTTACATGCCAATGTCTT
H-F	
GgbAS1	AATCTATGATAAGCCCATTGTTTTGCAGGAT
M256QC258AC260	GCATTGGCAAGAAACTAG
H-R	
GgbAS1	CCTGCAAAAGCTTGGGTTTATCATAGATTG
M256AC258VC260	GTTTACATGCCAATGTCTT
H-F	
GgbAS1	AATCTATGATAAACCCAAGCTTTTGCAGGA
M256AC258VC260	TGCATTGGCAAGAAACTAG
H-R	
GgbAS1	CCTGCAAAAGCTTGGGCTTATCATAGATTG
M256AC258AC260	GTTTACATGCCAATGTCTT
H-F	
GgbAS1	AATCTATGATAAGCCCAAGCTTTTGCAGGA
M256AC258AC260	TGCATTGGCAAGAAACTAG
H-R	

OEW M256AF	CCCTGGTAAAGCTTTGTGTTACTGCAGATT AG
OEW M256AR	CTGCAGTAACACAAAGCTTTACCAGGGTG GATTGG
OEW M256GF	CCCTGGTAAAGGTTTGTGTTACTGCAGATT AG
OEW M256GR	CTGCAGTAACACAAACCTTTACCAGGGTG GATTGG
OEW M256IF	CCCTGGTAAAATTTTGTGTTACTGCAGATTA G
OEW M256IR	CTGCAGTAACACAAAATTTTACCAGGGTGG ATTGG
OEW M256LF	CCCTGGTAAACTTTTGTGTTACTGCAGATT AG
OEWM259- RLR	CTGCAGTAACACAAAAGTTTACCAGGGTG GATTGG
OEW M256PF	CCCTGGTAAACCTTTGTGTTACTGCAGATT AG
OEW M256PR	CTGCAGTAACACAAAGGTTTACCAGGGTG GATTGG
OEW M256VF	CCCTGGTAAAGTTTTGTGTTACTGCAGATT AG
OEW M256VR	CTGCAGTAACACAAAACTTTACCAGGGTG GATTGG
OEW M256FF	CCCTGGTAAATTTTTGTGTTACTGCAGATTA G

OEW M256FR	CTGCAGTAACACAAAAATTTACCAGGGTG GATTGG
OEW M256WF	CCCTGGTAAATGGTTGTGTTACTGCAGATT AG
OEW M256WR	CTGCAGTAACACAACCATTTACCAGGGTGG ATTGG
OEW M256YF	CCCTGGTAAATATTTGTGTTACTGCAGATTA G
OEW M256YR	CTGCAGTAACACAAATATTTACCAGGGTGG ATTGG
OEW M256DF	CCCTGGTAAAGATTTGTGTTACTGCAGATT AG
OEW M256DR	CTGCAGTAACACAAATCTTTACCAGGGTGG ATTGG
OEW M256EF	CCCTGGTAAAGAATTGTGTTACTGCAGATT AG
OEW M256ER	CTGCAGTAACACAATTCTTTACCAGGGTGG ATTGG
OEW M256RF	CCCTGGTAAACGTTTGTGTTACTGCAGATT AG
OEW M256RR	CTGCAGTAACACAAACGTTTACCAGGGTG GATTGG
OEW M256HF	CCCTGGTAAACATTTGTGTTACTGCAGATT AG
OEW M256HR	CTGCAGTAACACAAATGTTTACCAGGGTGG ATTGG

OEW M256KF	CCCTGGTAAAAAATTGTGTTACTGCAGATT AG
OEW M256KR	CTGCAGTAACACAATTTTTTACCAGGGTGG ATTGG
OEW M256SF	CCCTGGTAAATCTTTGTGTTACTGCAGATTA G
OEW M256SR	CTGCAGTAACACAAAGATTTACCAGGGTG GATTGG
OEW M256TF	CCCTGGTAAAACTTTGTGTTACTGCAGATT AG
OEW M256TR	CTGCAGTAACACAAAGTTTTACCAGGGTG GATTGG
OEW M256CF	CCCTGGTAAATGTTTGTGTTACTGCAGATTA G
OEW M256CR	CTGCAGTAACACAAACATTTACCAGGGTG GATTGG
OEW M256NF	CCCTGGTAAAAATTTGTGTTACTGCAGATT AG
OEW M256NR	CTGCAGTAACACAAATTTTTACCAGGGTGG ATTGG
OEW M256QF	CCCTGGTAAACAATTGTGTTACTGCAGATT AG
OEW M256QR	CTGCAGTAACACAATTGTTTACCAGGGTGG ATTGG
OEWC258AF	GTAAAATGTTGGCTTACTGCAGATTAGTTT ACATGC

OEWC258AR	CTAATCTGCAGTAAGCCAACATTTTACCAG GGTG
OEWC258GF	GTAAAATGTTGGGTTACTGCAGATTAGTTT ACATGC
OEWC258GR	CACCCTGGTAAAATGTTGGGTTACTGCAGA TTAG
OEWC258IF	GTAAAATGTTGATTTACTGCAGATTAGTTTA CATGC
OEWC258IR	CTAATCTGCAGTAAATCAACATTTTACCAG GGTG
OEWC2580LF	GTAAAATGTTGCTTTACTGCAGATTAGTTTA CATGC
OEWC258LR	CACCCTGGTAAAATGTTGCTTTACTGCAGA TTAG
OEWC258PF	GTAAAATGTTGCCTTACTGCAGATTAGTTTA CATGC
OEWC258PR	CACCCTGGTAAAATGTTGCCTTACTGCAGA TTAG
OEWC258VF	GTAAAATGTTGGTTTACTGCAGATTAGTTTA CATGC
OEWC258VR	CTAATCTGCAGTAAACCAACATTTTACCAG GGTG
OEWC258FF	GTAAAATGTTGTTTTACTGCAGATTAGTTTA CATGC
OEWC258FR	CACCCTGGTAAAATGTTGTTTTACTGCAGA TTAG

OEWC258WF	GTAAAATGTTGTGGTACTGCAGATTAGTTTACATGC
OEWC258WR	CACCCTGGTAAAATGTTGTGGTACTGCAGATTAG
OEWC258YF	GTAAAATGTTGTATTACTGCAGATTAGTTTACATGC
OEWC258YR	CACCCTGGTAAAATGTTGTATTACTGCAGATTAG
OEWC258DF	GTAAAATGTTGGATTACTGCAGATTAGTTTACATGC
OEWC258DR	CACCCTGGTAAAATGTTGGATTACTGCAGATTAG
OEWC258EF	GTAAAATGTTGGAATACTGCAGATTAGTTTACATGC
OEWC258ER	CACCCTGGTAAAATGTTGGAATACTGCAGATTAG
OEWC258RF	GTAAAATGTTGCGTTACTGCAGATTAGTTTACATGC
OEWC258RR	CACCCTGGTAAAATGTTGCGTTACTGCAGATTAG
OEWC258HF	GTAAAATGTTGCATTACTGCAGATTAGTTTACATGC
OEWC258HR	CACCCTGGTAAAATGTTGCATTACTGCAGATTAG
OEWC258KF	GTAAAATGTTGAAATACTGCAGATTAGTTTACATGC

OEWC258KR	CACCCTGGTAAAATGTTGAAATACTGCAGA<br>TTAG
OEWC258SF	GTAAAATGTTGTCTTACTGCAGATTAGTTTA<br>CATGC
OEWC258SR	CACCCTGGTAAAATGTTGTCTTACTGCAGA<br>TTAG
OEWC258TF	GTAAAATGTTGACTTACTGCAGATTAGTTT<br>ACATGC
OEWC258TR	CACCCTGGTAAAATGTTGACTTACTGCAGA<br>TTAG
OEWC258MF	GTAAAATGTTGATGTACTGCAGATTAGTTTA<br>CATGC
OEWC258MR	CACCCTGGTAAAATGTTGATGTACTGCAGA<br>TTAG
OEWC258NF	GTAAAATGTTGAATTACTGCAGATTAGTTTA<br>CATGC
OEWC258NR	CACCCTGGTAAAATGTTGAATTACTGCAGA<br>TTAG
OEWC258QF	GTAAAATGTTGCAATACTGCAGATTAGTTT<br>ACATGC
OEWC258QR	CACCCTGGTAAAATGTTGCAATACTGCAGA<br>TTAG
OEWC260AF	GTTGTGTTACGCTAGATTAGTTTACATGCC
OEWC260AR	GTAAACTAATCTAGCGTAACACAACATTTT<br>AC

OEWC260GF	GTTGTGTTACGGTAGATTAGTTTACATGCC
OEWC260GR	GTAAACTAATCTACCGTAACACAACATTTT AC
OEWC260IF	:GTTGTGTTACATTAGATTAGTTTACATGCC
OEWC260IR	GTAAACTAATCTAATGTAACACAACATTTTA C
OEWC260LF	GTTGTGTTACCTTAGATTAGTTTACATGCC
OEWC260LR	GTAAACTAATCTAAGGTAACACAACATTTT AC
OEWC260PF	GTTGTGTTACCCTAGATTAGTTTACATGCC
OEWC260PR	GTAAACTAATCTAGGGTAACACAACATTTT AC
OEWC260FF	GTTGTGTTACTTTAGATTAGTTTACATGCC
OEWC260FR	GTAAACTAATCTAAAGTAACACAACATTTT AC
OEWC260WF	GTTGTGTTACTGGAGATTAGTTTACATGCC
OEWC260WR	GTAAACTAATCTCCAGTAACACAACATTTT AC
OEWC260YF	GTTGTGTTACTATAGATTAGTTTACATGCC

OEWC260YR	GTAAACTAATCTATAGTAACACAACATTTTA<br>C
OEWC260DF	GTTGTGTTACGATAGATTAGTTTACATGCC
OEWC260DR	GTAAACTAATCTATCGTAACACAACATTTTA<br>C
OEWC260EF	GTTGTGTTACGAAAGATTAGTTTACATGCC
OEWC260ER	GTAAACTAATCTTTCGTAACACAACATTTTA<br>C
OEWC260RF	GTTGTGTTACCGTAGATTAGTTTACATGCC
OEWC260RR	GTAAACTAATCTACGGTAACACAACATTTT<br>AC
OEWC260HF	GTTGTGTTACCATAGATTAGTTTACATGCC
OEWC260HR	GTAAACTAATCTATGGTAACACAACATTTTA<br>C
OEWC260KF	GTTGTGTTACAAAAGATTAGTTTACATGCC
OEWC260KR	GTAAACTAATCTTTTGTAACACAACATTTTA<br>C
OEWC260SF	GTTGTGTTACTCTAGATTAGTTTACATGCC
OEWC260SR	GTAAACTAATCTAGAGTAACACAACATTTT<br>AC

OEWC260TF	GTTGTGTTACACTAGATTAGTTTACATGCC	
OEWC260TR	GTAAACTAATCTAGTGTAACACAACATTTTAC	
OEWC260MF	GTTGTGTTACATGAGATTAGTTTACATGCC	
OEWC260MR	GTAAACTAATCTCATGTAACACAACATTTTAC	
OEWC260NF	GTTGTGTTACAATAGATTAGTTTACATGCC	
OEWC260NR	GTAAAATGTTGTGTTACAATAGATTAGTTTAC	
OEWC260QF	GTTGTGTTACCAAAGATTAGTTTACATGCC	3
OEWC260QR	GTAAACTAATCTTTGGTAACACAACATTTTAC	
OEWC260VF	GTTGTGTTACGTTAGATTAGTTTACATGCC	
OEWC260VR	GTAAACTAATCTAACGTAACACAACATTTTAC	
OEWM256LC260A-F	TAAACTTTTGTGTTACGCTAGATTAGTTTACATGCCAATGAGTTATTTG	
OEWM256LC260A-R	TCTAGCGTAACACAAAAGTTTACCAGGGTGGATTGGTGAG	
OEWM256VC260A-F	TAAAGTTTTGTGTTACGCTAGATTAGTTTACATGCCAATGAGTTATTTG	

OEWM256VC260A-R	TCTAGCGTAACACAAAACTTTACCAGGGTGGATTGGTGAG
OEWM256IC260A-F	TAAAATTTTGTGTTACGCTAGATTAGTTTACATGCCAATGAGTTATTTG
OEWM256IC260A-R	TCTAGCGTAACACAAAATTTTACCAGGGTGGATTGGTGAG
OEWM256YC260A-F	TAAATATTTGTGTTACGCTAGATTAGTTTACATGCCAATGAGTTATTTG
OEWM256YC260A-R	TCTAGCGTAACACAAATATTTACCAGGGTGGATTGGTGAG
OEWM256CC260A-F	TAAATGTTTGTGTTACGCTAGATTAGTTTACATGCCAATGAGTTATTTG
OEWM256CC260A-R	TCTAGCGTAACACAAACATTTACCAGGGTGGATTGGTGAG
OEWM256CC260G-F	TAAATGTTTGTGTTACGGTAGATTAGTTTACATGCCAATGAGTTATTTG
OEWM256CC260G-R	TCTACCGTAACACAAACATTTACCAGGGTGGATTGGTGAG
OEWM256LC260G-F	TAAACTTTTGTGTTACGGTAGATTAGTTTACATGCCAATGAGTTATTTG
OEWM256LC260G-R	TCTACCGTAACACAAAAGTTTACCAGGGTGGATTGGTGAG
OEWM256VC260G-F	TAAAGTTTTGTGTTACGGTAGATTAGTTTACATGCCAATGAGTTATTTG
OEWM256VC260G-R	TCTACCGTAACACAAAACTTTACCAGGGTGGATTGGTGAG

OEWM256IC260G-F	TAAAATTTTGTGTTACGGTAGATTAGTTTACATGCCAATGAGTTATTTG
OEWM256IC260G-R	TCTACCGTAACACAAAATTTTACCAGGGTGGATTGGTGAG
OEWM256YC260G-F	TAAATATTTGTGTTACGGTAGATTAGTTTACATGCCAATGAGTTATTTG
OEWM256YC260G-R	TCTACCGTAACACAAATATTTACCAGGGTGGATTGGTGAG
MdOSC1 M256AR	GTAACAAAAAGCTTTAGATGGATGGAAgtgTAACAATGTTG
MdOSC1 M256GF	CCATCCATCTAAAGGTTTTTGTTACTGCAGATTAACTTATTTGCC
MdOSC1 M256GR	GTAACAAAAACCTTTAGATGGATGGAATGGTAACAATGTTG
MdOSC1 M256IF	CCATCCATCTAAAATTTTTTGTTACTGCAGATTAACTTATTTGCC
MdOSC1 M256IR	GTAACAAAAAATTTTAGATGGATGGAATGGTAACAATGTTG
MdOSC1 M256LF	CCATCCATCTAAACTTTTTTGTTACTGCAGATTAACTTATTTGCC
MdOSC1 M256LR	GTAACAAAAAAGTTTAGATGGATGGAATGGTAACAATGTTG
MdOSC1 M256PF	CCATCCATCTAAACCTTTTTGTTACTGCAGATTAACTTATTTGCC
MdOSC1 M256PR	GTAACAAAAAGGTTTAGATGGATGGAATGGTAACAATGTTG

MdOSC1 M256VF	GTAACAAAAAGGTTTAGATGGATGGAATG GTAACAATGTTG
MdOSC1 M256VR	GTAACAAAAAACTTTAGATGGATGGAATGG TAACAATGTTG
MdOSC1 M256FF	CCATCCATCTAAATTTTTTTGTTACTGCAGA TTAACTTATTTGCC
MdOSC1 M256FR	GTAACAAAAAAATTTAGATGGATGGAATGG TAACAATGTTG
MdOSC1 M256WF	CCATCCATCTAAATGGTTTTGTTACTGCAGA TTAACTTATTTGCC
MdOSC1 M256WR	GTAACAAAACCATTTAGATGGATGGAATGG TAACAATGTTG
MdOSC1 M256YF	CCATCCATCTAAATATTTTTGTTACTGCAGA TTAACTTATTTGCC
MdOSC1 M256YR	GTAACAAAAATATTTAGATGGATGGAATGG TAACAATGTTG
MdOSC1 M256DF	CCATCCATCTAAAGATTTTTGTTACTGCAGA TTAACTTATTTGCC
MdOSC1 M256DR	GTAACAAAAATCTTTAGATGGATGGAATGG TAACAATGTTG
MdOSC1 M256EF	CCATCCATCTAAAGAATTTTGTTACTGCAG ATTAACTTATTTGCC
MdOSC1 M256ER	GTAACAAAATTCTTTAGATGGATGGAATGG TAACAATGTTG
MdOSC1 M256RF	CCATCCATCTAAACGTTTTTGTTACTGCAG ATTAACTTATTTGCC

MdOSC1 M256RR	GTAACAAAAACGTTTAGATGGATGGAATGG TAACAATGTTG
MdOSC1 M256HF	CCATCCATCTAAACATTTTTGTTACTGCAGA TTAACTTATTTGCC
MdOSC1 M256HR	GTAACAAAAATGTTTAGATGGATGGAATGG TAACAATGTTG
MdOSC1 M256KF	CCATCCATCTAAAAAATTTTGTTACTGCAG ATTAACTTATTTGCC
MdOSC1 M256KR	GTAACAAAATTTTTTAGATGGATGGAATGG TAACAATGTTG
MdOSC1 M256SF	CCATCCATCTAAATCTTTTTGTTACTGCAGA TTAACTTATTTGCC
MdOSC1 M256SR	GTAACAAAAAGATTTAGATGGATGGAATGG TAACAATGTTG
MdOSC1 M256TF	CCATCCATCTAAAACTTTTTGTTACTGCAG ATTAACTTATTTGCC
MdOSC1 M256TR	GTAACAAAAAGTTTAGATGGATGGAATGG TAACAATGTTG
MdOSC1 M256CF	CCATCCATCTAAATGTTTTTGTTACTGCAGA TTAACTTATTTGCC
MdOSC1 M256CR	GTAACAAAAACATTTAGATGGATGGAATGG TAACAATGTTG
MdOSC1 M256NF	CCATCCATCTAAAAATTTTTGTTACTGCAGA TTAACTTATTTGCC
MdOSC1 M256NR	GTAACAAAAATTTTTAGATGGATGGAATGG TAACAATGTTG

MdOSC1 M256QF	CCATCCATCTAAACAATTTTGTTACTGCAG ATTAACTTATTTGCC
MdOSC1 M256QR	GTAACAAAATTGTTTAGATGGATGGAATGG TAACAATGTTG
MdOSC1 C258AF	CTAAAATGTTTGCTTACTGCAGATTAACTTA TTTGCCAATG
MdOSC1 C258AR	AATCTGCAGTAAGCAAACATTTTAGATGGA TGGAATGGTAAC
MdOSC1 C258GF	CTAAAATGTTTGGTTACTGCAGATTAACTTA TTTGCCAATG
MdOSC1 C258GR	AATCTGCAGTAACCAAACATTTTAGATGGA TGGAATGGTAAC
MdOSC1 C258IF	CTAAAATGTTTATTTACTGCAGATTAACTTA TTTGCCAATG
MdOSC1 C258IR	AATCTGCAGTAAATAAACATTTTAGATGGAT GGAATGGTAAC
MdOSC1 C258LF	CTAAAATGTTTCTTTACTGCAGATTAACTTA TTTGCCAATG
MdOSC1 C258LR	AATCTGCAGTAAAGAAACATTTTAGATGGA TGGAATGGTAAC
MdOSC1 C258PF	CTAAAATGTTTCCTTACTGCAGATTAACTTA TTTGCCAATG
MdOSC1 C258PR	AATCTGCAGTAAGGAAACATTTTAGATGGA TGGAATGGTAAC
MdOSC1 C258VF	CTAAAATGTTTGTTTACTGCAGATTAACTTA TTTGCCAATG

MdOSC1 C258VR	AATCTGCAGTAAACAAACATTTTAGATGGA<br>TGGAATGGTAAC
MdOSC1 C258FF	CTAAAATGTTTTTTTACTGCAGATTAACTTA<br>TTTGCCAATG
MdOSC1 C258FR	AATCTGCAGTAAAAAAACATTTTAGATGGA<br>TGGAATGGTAAC
MdOSC1 C258WF	CTAAAATGTTTTGGTACTGCAGATTAACTTA<br>TTTGCCAATG
MdOSC1 C258WR	AATCTGCAGTACCAAAACATTTTAGATGGA<br>TGGAATGGTAAC
MdOSC1 C258YF	CTAAAATGTTTTATTACTGCAGATTAACTTA<br>TTTGCCAATG
MdOSC1 C258YR	AATCTGCAGTAATAAAACATTTTAGATGGAT<br>GGAATGGTAAC
MdOSC1 C258DF	CTAAAATGTTTGATTACTGCAGATTAACTTA<br>TTTGCCAATG
MdOSC1 C258DR	AATCTGCAGTAATCAAACATTTTAGATGGA<br>TGGAATGGTAAC
MdOSC1 C258EF	CTAAAATGTTTGAATACTGCAGATTAACTTA<br>TTTGCCAATG
MdOSC1 C258ER	AATCTGCAGTATTCAAACATTTTAGATGGAT<br>GGAATGGTAAC
MdOSC1 C258RF	CTAAAATGTTTCGTTACTGCAGATTAACTTA<br>TTTGCCAATG
MdOSC1 C258RR	AATCTGCAGTAACGAAACATTTTAGATGGA<br>TGGAATGGTAAC

MdOSC1 C258HF	CTAAAATGTTTCATTACTGCAGATTAACTTATTTGCCAATG
MdOSC1 C258HR	AATCTGCAGTAATGAAACATTTTAGATGGATGGAATGGTAAC
MdOSC1 C258KF	CTAAAATGTTTAAATACTGCAGATTAACTTATTTGCCAATG
MdOSC1 C258KR	AATCTGCAGTATTTAAACATTTTAGATGGATGGAATGGTAAC
MdOSC1 C258SF	CTAAAATGTTTTCTTACTGCAGATTAACTTATTTGCCAATG
MdOSC1 C258SR	AATCTGCAGTAAGAAAACATTTTAGATGGATGGAATGGTAAC
MdOSC1 C258TF	CTAAAATGTTTACTTACTGCAGATTAACTTATTTGCCAATG
MdOSC1 C258TR	AATCTGCAGTAAGTAAACATTTTAGATGGATGGAATGGTAAC
MdOSC1 C258MF	CTAAAATGTTTATGTACTGCAGATTAACTTATTTGCCAATG
MdOSC1 C258MR	AATCTGCAGTACATAAACATTTTAGATGGATGGAATGGTAAC
MdOSC1 C258NF	CTAAAATGTTTAATTACTGCAGATTAACTTATTTGCCAATG
MdOSC1 C258NR	AATCTGCAGTAATTAAACATTTTAGATGGATGGAATGGTAAC
MdOSC1 C258QF	CTAAAATGTTTCAATACTGCAGATTAACTTATTTGCCAATG

MdOSC1 C258QR	AATCTGCAGTATTGAAACATTTTAGATGGATGGAATGGTAAC
MdOSC1 C260AF	ATGTTTTGTTACGCTAGATTAACTTATTTGCCAATGTCTT
MdOSC1 C260AR	AAGTTAATCTAGCGTAACAAAACATTTTAGATGGATGG
MdOSC1 C260GF	ATGTTTTGTTACGGTAGATTAACTTATTTGCCAATGTCTT
MdOSC1 C260GR	AAGTTAATCTACCGTAACAAAACATTTTAGATGGATGG
MdOSC1 C260IF	ATGTTTTGTTACATTAGATTAACTTATTTGCCAATGTCTT
MdOSC1 C260IR	AAGTTAATCTAATGTAACAAAACATTTTAGATGGATGG
MdOSC1 C260LF	ATGTTTTGTTACCTTAGATTAACTTATTTGCCAATGTCTT
MdOSC1 C260LR	AAGTTAATCTAAGGTAACAAAACATTTTAGATGGATGG
MdOSC1 C260PF	ATGTTTTGTTACCCTAGATTAACTTATTTGCCAATGTCTT
MdOSC1 C260PR	AAGTTAATCTAGGGTAACAAAACATTTTAGATGGATGG
MdOSC1 C260VF	ATGTTTTGTTACGTTAGATTAACTTATTTGCCAATGTCTT
MdOSC1 C260VR	AAGTTAATCTAACGTAACAAAACATTTTAGATGGATGG

MdOSC1 C260FF	ATGTTTTGTTACTTTAGATTAACTTATTTGC CAATGTCTT
MdOSC1 C260FR	AAGTTAATCTAAAGTAACAAAACATTTTAG ATGGATGG
MdOSC1 C260WF	ATGTTTTGTTACTGGAGATTAACTTATTTGC CAATGTCTT
MdOSC1 C260WR	AAGTTAATCTCCAGTAACAAAACATTTTAG ATGGATGG
MdOSC1 C260YF	ATGTTTTGTTACTATAGATTAACTTATTTGCC AATGTCTT
MdOSC1 C260YR	AAGTTAATCTATAGTAACAAAACATTTTAG ATGGATGG
MdOSC1 C260DF	ATGTTTTGTTACGATAGATTAACTTATTTGC CAATGTCTT
MdOSC1 C260DR	AAGTTAATCTATCGTAACAAAACATTTTAG ATGGATGG
MdOSC1 C260EF	ATGTTTTGTTACGAAAGATTAACTTATTTGC CAATGTCTT
MdOSC1 C260ER	AAGTTAATCTTTCGTAACAAAACATTTTAG ATGGATGG
MdOSC1 C260RF	ATGTTTTGTTACCGTAGATTAACTTATTTGC CAATGTCTT
MdOSC1 C260RR	AAGTTAATCTACGGTAACAAAACATTTTAG ATGGATGG
MdOSC1 C260HF	ATGTTTTGTTACCATAGATTAACTTATTTGC CAATGTCTT

MdOSC1 C260HR	AAGTTAATCTATGGTAACAAAACATTTTAG ATGGATGG
MdOSC1 C260KF	ATGTTTTGTTACAAAAGATTAACTTATTTGC CAATGTCTT
MdOSC1 C260KR	AAGTTAATCTTTTGTAACAAAACATTTTAG ATGGATGG
MdOSC1 C260SF	ATGTTTTGTTACTCTAGATTAACTTATTTGC CAATGTCTT
MdOSC1 C260SR	AAGTTAATCTAGAGTAACAAAACATTTTAG ATGGATGG
MdOSC1 C260TF	ATGTTTTGTTACACTAGATTAACTTATTTGC CAATGTCTT
MdOSC1 C260TR	AAGTTAATCTAGTGTAACAAAACATTTTAG ATGGATGG
MdOSC1 C260MF	ATGTTTTGTTACATGAGATTAACTTATTTGC CAATGTCTT
MdOSC1 C260MR	AAGTTAATCTCATGTAACAAAACATTTTAG ATGGATGG
MdOSC1 C260NF	ATGTTTTGTTACAATAGATTAACTTATTTGC CAATGTCTT
MdOSC1 C260NR	AAGTTAATCTATTGTAACAAAACATTTTAG ATGGATGG
MdOSC1 C260QF	ATGTTTTGTTACCAAAGATTAACTTATTTGC CAATGTCTT
MdOSC1 C260QR	AAGTTAATCTTTGGTAACAAAACATTTTAG ATGGATGG

Reference

Dai, Z., Wang, B., Liu, Y., Shi, M., Wang, D., Zhang, X., Liu, T., Huang, L., Zhang, X., 2014. Producing aglycons of ginsenosides in bakers yeast. Sci Rep. 4 , 3698-3698.

Jensen, N. B., Strucko, T., Kildegaard, K. R., David, F., Maury, J., Mortensen, U. H., Forster, J., Nielsen, J., Borodina, I., 2014. EasyClone: method for iterative chromosomal integration of multiple genes in *Saccharomyces cerevisiae*. FEMS Yeast Res. 14, 238-48.
